# Supplementary material for: Impact of vaccination against Japanese encephalitis in endemic countries
Source: PLoS Negl Trop Dis. 2024 Sep 3;18(9):e0012390. doi: 10.1371/journal.pntd.0012390 (PMC11398676; doi:10.1371/journal.pntd.0012390)
Supplement: S1 Appendix — (DOCX) [file pntd.0012390.s001.docx]

**Appendix 1. Surveillance type, immunization program approach, vaccine used, comparative periods, vaccination coverage, and data sources for countries introducing or expanding Japanese encephalitis (JE) vaccination in 2006 or later**

| ***Country*** | ***Surveillance type*** | ***Immunization program during analysis periods*** | ***Vaccine and schedule*** | ***Periods of analysis*** | ***Vaccination coverage*** | ***Data sources*** | ***Notes*** |
| --- | --- | --- | --- | --- | --- | --- | --- |
| Cambodia | Meningoencephalitis surveillance with JE IgM testing among children ≤15 years of age | SIA:  2013: Subnational SIA for 9 months–12 years of age in 1 of ~24 provinces  2016: National SIA for 9 months–14 years of age (Q1)  RI:  2009–2012: Subnational RI for 10–23 months of age in 3 of ~24 provinces (commenced Oct 2009)  2013– 2014: No RI  2015: Subnational RI for 10–12 months of age in 6 of ~24 provinces  2016: National RI for 10–12 months of age (began following 2016 SIA) | CD-JEV  Campaign (SIA): single dose  RI: single dose | 2007–2015 (9 years) vs. 2016–2018 (3 years) | SIA:  2013: 96% (subnational SIA in 1 province)  2016: 102% (national SIA)  RI:  2009–2015: Average 70% (Range: 0%–140%)  2016–2018: Average 59% (Range 14%–91%) | Cambodian MOH (WHO Protocol-Survey data)[6] | Comparison is between earlier period with subnational program and later period with national program  No data provided for 2006  Coverage rates >100% might be due to underestimates of target population, vaccine catch-up programs, and/or vaccination of children from non-target areas  RI coverage of 0% in 2013 and 2014 and 14% in 2017 due to vaccine stock outs  Number of provinces in Cambodia has changed slightly over time |
| India (Uttar Pradesh State) | AES surveillance (WHO definition)*  and JE IgM testing for all ages  District surveillance sites increased over time, selected for testing of AES cases with JE IgM based on risk assessment for JE virus transmission and intention to introduce JE vaccine in near future[1] | SIA:  Introduction by year based on risk assessment, starting with highest risk of 75 districts in UP  2006: 7 highest risk districts  2007: 12 districts  2008: 10 districts  2009: 7 districts  2010: Repeat SIA in initial 7 districts (from 2006)  2014: 1 district  2015: 2 districts  RI: For 10–12 months of age.  2007–2008: 1 district  2009–2016: 34–38 districts | CD-JEV  SIA: single dose  RI:  2007: single dose  2008–2018: 2 doses | 2005–11 (7 years) vs.  2012–18  (7 years) | SIAs:  Average 96%  RI:  2007–2011 Average: 26%  (Range 2–47%)  2012–18:  Average: 50%  (Range 19%–70%) | (WHO protocol-Survey data) [6] and published data by Indian/UP MOH[1] | Uttar Pradesh accounted for increasing proportion of JE cases reported in India over time: ~20% 1978–1998; ~40% 1998–2004; 75–80% 2005–2009 [2]. For this reason, JE vaccine impact in Uttar Pradesh (comparing even number of years 2005–18) approximates JE immunization impact in India overall [2]. |
| Indonesia (Bali Province only) | Acute encephalitis/meningitis/AFP surveillance and JE IgM testing among children <15 years of age | SIA:  2018: Provincial SIA for 9 months–15 years of age (March-April)  RI:  2018: Provincial RI for 10 months of age commencing 4–8 weeks after SIA | CD-JEV  Campaign (SIA): single dose  RI: single dose | 2014–2017 (4 years) vs. 2018–2021 (4 years) | SIA  2018: 94% (SIA)  RI  2018–2021: Average 64% (Range: 53%– 76%) | Indonesian MOH (WHO Protocol- Survey data)[6]  Bali Provincial Health Department [3]  Published data [4] | Comparison between pre-vaccine and post vaccine periods  Vaccination was in all districts of Bali province |
| Laos | AES surveillance (WHO definition)*  and JE IgM testing for all ages | SIA:  2013: Subnational SIA for 1–14 years of age in 6 of 18 provinces  2014: Subnational SIA for 1–14 years of age in additional 2 provinces  2015: Subnational SIA (in April) for children 1–14 years of age in remaining 10 18 provinces  RI:  2016: National RI for 9–11 months of age | CD-JEV  Campaign (SIA): single dose  RI: single dose | 2010–2014 (5 years) vs. 2015–2018 (4 years) | SIA  2013–2015: By 2015, 95% all children 1–14 years of age had been vaccinated in a SIA  RI  2016–2018: Average 55% (Range: 44%–70%) | Laos MOH (WHO Protocol-Survey data)[6] | Periods chosen to demonstrate impact after nationwide implementation of vaccination for children aged <15 years i.e., comparison between earlier period with 8 subnational SIAs (i.e., 6 in 2013 and 2 in 2014) and no RI with later period which included completion of SIAs in remaining 10 provinces in 2015 (which meant 95% children aged 1–14 years had been vaccinated in a SIA), and establishment of national RI in 2016 |
| Myanmar | AES surveillance (WHO definition)* and JE IgM testing for all ages | SIA:  2017: National SIA (in November-December) for 9 months–15 years of age  RI:  2018: National RI at 9 months of age (commencing January-February) | CD-JEV  Campaign (SIA): single dose  RI: single dose | 2015–2017 (3 years) vs. 2018–2020 (3 years) | SIA  2017: 93%  RI  2018–2020: Average 87% (Range: 84%–89%) | Myanmar MOH and WHO country representative (WHO protocol-Survey data[6]  Published data [5] | Comparison between pre-vaccine and post vaccine periods.  Myanmar MOH had highest confidence in data by 2015, so pre-vaccine period began 2015 |
| Nepal | AES surveillance (WHO definition)* and JE IgM testing for all ages | SIA:  2006–2011: Subnational SIA in 31 of 75 administrative districts with high or moderate JE risk. Implemented among all persons ≥1 year in 20 districts and children 1–15 years of age in 11 districts  2016: Subnational SIA for 1–15 years of age in 44 remaining lower risk districts  RI:  2010–2011: Subnational RI for 12–23 months of age in 22 districts  2012–16: Subnational RI for 12–23 months of age in total of 31 districts  2017: National RI for 12 months of age | CD-JEV  Campaign (SIA): single dose  RI: single dose | 2006–2011 (6 years)  vs.  2012–2018 (7 years) | SIA  2006–2011: Average: 88% (Range: 75%–97%)  2016: 106%  RI  2010–2011: Average: 50% (Range: 47%–54%)  2012–2018: Average: 71% (Range: 62%–79%) | Nepal MOH and WHO country representative (WHO Protocol-Survey data)[6] | Comparison of period when vaccination program was introduced in high and moderate risk districts and period with gradual expansion to lower risk districts and establishment of comprehensive national program  Coverage rate higher  than 100% might have been due to residents of other districts being vaccinated and included  in counts or underestimates of a district's population |
| *World Health Organization acute encephalitis syndrome (AES) surveillance case definition: A person with the acute onset of fever and at least one of the following: a) a change in mental status (including symptoms such as confusion, disorientation, coma or inability to talk) or b) new onset of seizures (excluding simple febrile seizures). (Available at: <https://www.who.int/teams/immunization-vaccines-and-biologicals/immunization-analysis-and-insights/surveillance/surveillance-for-vpds/vpd-surveillance-standards>)  1. Singh AK, Kariya P, Agarwal V, Singh S, Singh NP, Jain PK, Kumar S, Bajpai SK, Dixit AM, Singh RK, Agarwal T. Japanese encephalitis in Uttar Pradesh, India: A situational analysis. J Family Med Prim Care 2020;9:3716-21.  2. Kumari R, Joshi PL. A review of Japanese encephalitis in Uttar Pradesh, India. WHO South-East Asia J of Public Health. 2012;1(4):374-95.  3. Bali Provincial Health Office (Dinas Kesehatan Provinsi Bali). Laporan Tahunan Japanese Encephalitis 2015-2019. Published online 2020.  4. Sawitri AAS, Yuliatni PCD, Ariawan MD, Sari KAK, Susanti R, Sutarsa IN. Limitations of immunization registers at community health centers for measuring immunization coverage: a case study of the Japanese encephalitis mass immunization program in Bali Province, Indonesia. Osong Public Health Res Perspect 2021: <https://doi.org/10.24171/j.phrp.2020.024>  5. Win AYN, Wai KT, Harries AD, Kyaw NTT, Oo T, Than WP, Lin HH, Lin Z. The burden of Japanese encephalitis, the catch up vaccination campaign, and health provider’s perceptions in Myanmar: 2012-2017. Trop Med Health 2020;48:13.  [https://doi.org/10.1186/s41182-020-00200-3](https://doi.org/10.1186/s41182-020-00200-3 6)  [6](https://doi.org/10.1186/s41182-020-00200-3 6). S1 Raw-Data WHO-PATH protocol file | | | | | | | |
| Abbreviations:  AES: acute encephalitis syndrome  AFP: acute flaccid paralysis  CD-JEV: live, attenuated SA14-14-2 JE vaccine  IgM: immunoglobulin M  MOH: Ministry of Health  Q: quarter  RI: routine immunization  SIA: supplementary immunization activity  WHO: World Health Organization | | | | | | | |
