# Supplementary material for: Impact of vaccination against Japanese encephalitis in endemic countries
Source: PLoS Negl Trop Dis. 2024 Sep 3;18(9):e0012390. doi: 10.1371/journal.pntd.0012390 (PMC11398676; doi:10.1371/journal.pntd.0012390)
Supplement: S2 Appendix — (DOCX) [file pntd.0012390.s002.docx]

**Appendix 2. Surveillance type, immunization program approach, vaccine used, comparative periods, vaccination coverage, and data sources for countries introducing or expanding Japanese encephalitis (JE) vaccination before 2006.**

| ***Country*** | ***Surveillance type*** | ***Immunization program during analysis periods*** | ***Vaccine and schedule*** | ***Period of analysis*** | ***Vaccination Coverage*** | ***Data sources*** | ***Notes*** |
| --- | --- | --- | --- | --- | --- | --- | --- |
| Malaysia (Sarawak State) | Viral encephalitis surveillance and IgM testing for all ages | SIA:  Not done  RI:  2001: Statewide program for children aged 9 months | Mouse brain-derived inactivated vaccine (2001–2013): 2 doses 1 month apart (primary series) and subsequent booster(s) (schedule variable over time)  Chimeric vaccine (from 2014): 1 dose primary series and 1 booster (21 months of age) | 1996–2001 (6 years) vs.  2006–2015 (10 years)  (All ages for all of Maylasia and among children <15 years in Sarawak alone) | RI  2001–2008: NA  2009–2015: Average: 96% (Range: 90%–100%)(NB. Similar coverage rates for mouse brain-derived and chimeric vaccine) | Biregional data [1]  Published data [2] | Analysis focused on Sarawak which had a routine immunization program; in peninsular Malaysia, only targeted vaccination is used (e.g., outbreaks)  Comparison of period leading up to, and first year of, vaccine introduction and most recent 10-year period for which data are available |
| Sri Lanka | Encephalitis surveillance and IgM testing for all ages | SIA:  1988–2008: Subnational SIAs introduced in phased manner in 18 high risk districts among children aged 1–10 years  RI:  1988–2010:  Subnational in 18 high risk districts for 1 year of age (introduced following SIA in each district)  2011: National for children aged 9 months | Mouse brain-derived inactivated vaccine (1988–2008)  Campaign (SIA):  3-dose primary series (0, 1, 12 months) and 1 booster dose (4–5 years after dose 1)  RI: 3-dose primary series and 1 booster dose (schedule as above)  CD-JEV (from 2009)  RI: single dose | 1985–1987 (3 years) vs. 2013–2015 (3 years) (JE among all age groups) | SIAs  1988–2008: >90%  RI  2009–2016: >95% | Biregional data [1] | Comparison between 3-year pre-vaccine period and most recent 3-year period for which data available |
| Thailand | Encephalitis surveillance for all ages, and JE IgM testing at 40 sentinel sites with a proportion of samples tested | SIA: Not done  RI:  1990–1999:  Subnational program introduced in phased manner, initially in 8 provinces and ultimately in 34 higher risk provinces, for children 1.5 years of age  2000: National program for children at 1–1.5 years of age (variability in starting age by vaccine type)(2000–2012: Mouse brain-derived inactivated vaccine; 2013–2016 [April]: CD-JEV introduced, initially in 8 high risk provinces and ultimately in 29 provinces, and mouse brain-derived inactivated vaccine used in others; 2016 [May - December]: CD-JEV used nationally; 2017–2018: Live attenuated vaccines [CD-JEV and IMOJEV]) used nationally | Mouse-brain derived inactivated vaccine (1990–2016 [April]): 2 doses 1 month apart starting at 1.5 years of age (primary series) and 1 booster (2.5–3 years of age; booster introduced in 1994)  CD-JEV (from 2013): 1 dose primary series (1 year of age) and 1 booster (2.5 years of age)  IMOJEV (from 2017): 1 dose primary series (1 year of age) and 1 booster (2.5 years of age) | 1977–1989 (13 years) vs. 2011–2015 (5 years)(Encephalitis among all age groups) | RI  Coverage for primary series from coverage surveys  1999: 84%  2003: 87%  2008: 95%  2013: 96% | Biregional data [1]  Thailand MOH  (WHO Protocol-Survey data) | Comparison based on encephalitis data for population overall, analyzing pre-vaccine period and most recent 5-year period for which data available |
| Vietnam | Viral encephalitis surveillance for all ages and JE IgM testing with a proportion of samples tested  2006–2019: Sentinel population-based JE surveillance, initially in 3 provinces and ultimately by 2013 in 5 provinces  2009–2019: Sentinel meningoencephalitis surveillance, initially in 2 pediatric hospitals and ultimately by 2013 in 3 main pediatric hospitals in Hanoi and Ho Chi Minh city | SIA:  1997–2014:  Subnational SIAs introduced in phased manner, initially in 12 higher risk districts of about 680 districts and ultimately in 100% of districts. Program for children aged 1–5 years in first year, then for children 1 year of age in following years  2018: Subnational SIA for ages 6–15 years in 28 high risk districts in 16 provinces  RI  2015: National program for children 1 year of age | Mouse brain-derived inactivated vaccine: 2 doses 1–2 weeks apart (primary series) and 1 booster dose 1 year later | 1991–1996 (6 years) vs. 2015--2018 (4 years) (Encephalitis among all age groups) | SIA  1997–2014: Average 93% (range: 88%–99%)  2018: 96% (SIA in 28 districts)  RI  2015–2018: Average 93% (range: 91%–95%) | Biregional meeting [1]  Viet Nam MOH (WHO Protocol- Survey data)  Published data [3] | Comparison between period before JE vaccine program introduction and since program implemented in 100% of districts, using encephalitis data for all age groups |
| Japan | National encephalitis surveillance since 1965 for all age groups, with laboratory  confirmation initially by hemagglutination inhibition or complement fixation tests, and more recently by neutralization assay, polymerase chain reaction, and ELISA testing [4] | SIA:  Not done  RI:  1967: National program with vaccination commencing in early childhood  Recommendation for vaccination suspended from 2005–2008 when Japan stopped using mouse brain-derived vaccine, but reinstated in 2009 when Vero cell-derived vaccines became available | Mouse brain derived inactivated vaccine (1967–2005): 2 primary and 1 booster dose in early childhood, and 2 booster doses in older childhood/adolsecence  Vero cell-derived vaccines (from 2009): 3 dose in early childhood and 1 booster dose in older childhood/adolescence | 1950–1967 (18 years) vs. 1991–2015 (25 years)(JE among all age groups) | RI  Vaccine coverage 83–93% from 1996–2004, declined to 4% from 2005–9, and rose to ~100% in 2010–15 after switch from mouse brain to Vero cell-derived JE vaccine [5]. | Biregional meeting [1]  Published data [6,7] | Comparison of JE incidence in 18 years prior to, and year of, introduction of vaccination program and period since early 1990s when case numbers have consistently been <10 cases/year |
| People’s Republic of China | National encephalitis surveillance for all ages and IgM testing with a proportion of samples tested  2006: Sentinel meningitis/encephalitis surveillance in 4 cities of 4 provinces | RI  Late 1960s: Vaccine available for purchase. Vaccination programs subsequently implemented as possible by local governments. From 1980s, the number of programs increased with 16 provinces having programs by 2006  2008: JE vaccine included in the routine immunization program nationwide except non-endemic provinces of Xinjiang, Tibet and Qinghai provinces  SIA  2009: Subnational SIAs in 308 counties in 12 provinces with incidence >1/100,000 | Inactivated primary hamster kidney cell-derived (P3) vaccine (from 1968, no longer used by ~2010): Schedule variable over time but ultimately 4 doses with 2 doses at 8 months (interval of 7–10 days) and boosters at 2 years and 6 years of age  CD-JEV (from 1989): 2 doses at 8 months and 2 years of age (booster at 6 years of age discontinued)  Inactivated Vero cell-derived (P3) vaccine (from ~2004): 4 doses with 2 doses given at 8 months (interval of 7–10 days) and boosters at 2 years and 6 years of age | 1967–1970 (4 years) vs. 2010–2013 (4 years)(JE among all age groups) | RI  Determined by survey in 2013 was at least 95% with a few townships at <90%  Based on reported data in 2015 was at least 95% | Biregional data [1]  Published data [8,9] | Comparison based on period when vaccine use limited (only available for purchase) and period beginning in period after JE vaccine included in routine immunization program nationwide and subnational SIAs conducted |
| Chinese Taipei | JE has been a notifiable disease for all ages since 1955  Laboratory testing established in 1965 and active surveillance in 1967. Initially testing with hemagglutination inhibition tests and from 1998 by IgM ELISA [10]. | SIA:  Not done  RI:  1968: National program with vaccination commencing in early childhood | Mouse brain-derived inactivated vaccine (1968–2017): Initially two doses given two weeks apart to children aged ~ 2 years (age later lowered to 15 months). In 1974, booster added at 1 year after primary series. In 1976, second booster added at elementary school entry.  Began switch to chimeric vaccine in 2017 and used exclusively since 2018: 1 dose at 15 months and booster at 27 months | 1966–1968 (3 years) vs. 2008–12 (5 years) | RI  1971–1985: Second dose coverage ≥80%  1986 and onwards: ~90–95% | Chinese Taipei MOH (WHO protocol-Survey data)  Published data [10] | Comparison of pre-vaccination period/ first year of vaccination program and recent 5-year period |
| Republic of Korea | JE was a notifiable disease from 1949  National encephalitis surveillance with laboratory confirmation by IgM ELISA and other modalities [11-14]. | SIA:  1983: 3–15 years of age [15]  RI:  1967: Vaccine available nationwide  1983: Vaccination part of national immunization program | Mouse brain-derived inactivated vaccine (from 1967) with first dose at 3 years, 2^nd^ dose one month later, and third one year later with booster every year until age 15 years, and subsequently (1995–1999) booster recommended every other year. Later (2000) primary series begun at 1–2 years of age with boosters at 6 and 12 years  CD-JEV (2014): 2 doses at 12 month interval  Vero cell-derived JE vaccines (2015): 2 doses at 7–30 day interval, boosters 12 months and 6 and 12 years later | 1960–1966 (7 years) vs. 2006–2018 (13 years) for under 15 years and all ages | RI  1970s: <5%  1981: 17%  1983: 60%  1985–1992:~90%  2006–2018: 99% | Biregional data [1]  Published data [12,13] | Comparison of period prior to vaccine availability and recent 13-year period |
| 1. WHO. Regional Office for Western Pacific. Meeting report of the seventh biregional meeting on prevention and control of Japanese Encephalitis. Presented at Manila, Philippines, 2016. <http://www.wpro.who.int/entity/immunization/documents/prevention_and_control_of_je.pdf?ua=1> 2. Kumar K, Arshad SS, Selvaraja GT, Abu J, Toung OP, Abba Y, Yasmin AR, Bande F, Sharma R, Ong BL. Japanese encephalitis in Malaysia: An overview and timeline. Acta Tropica 2018;185:219-29. 3. Yen NT, Duffy MR, Hong NM, Tien NT, Fischer M, Hills SL. Surveillance for Japanese encephalitis in Vietnam, 1998-2007. Am J Trop Med Hyg 2010;83:816-19. 4. Arai S, Matsunaga Y, Takashi T, Tanaka-Taya K, Taniguchi K, Okabe N, Kurane I. Japanese encephalitis: Surveillance and elimination effort in Japan from 1982-2004. Jpn J Infect Dis 2008;61:333-8. 5. Nanishi E, Hoshina T, Sanefuji M, Kadoya R, Kitazawa K, Arahat Y, Sato T, Hirayama Y, Hirai K, Yanai M, Nikaido K, Maeda A, Torisu H, Okada K, Sakai Y, Ohga S. A national survey of pediatric-onset Japanese encephalitis in Japan. Clin Infect Dis 2019;68(12);2099-2104. 6. Konishi E, Kitai Y, Tabei Y, Nishimura K, Harada S. Natural Japanese encephalitis virus infection among humans in west and east Japan shows the need to continue a vaccination program. Vaccine 2010;28:2664-70. 7. Igarashi A. Control of Japanese encephalitis in Japan: Immunization of humans and animals and vector control. Curr Top Micobiol Immunol 2002;267:139-152. 8. Yu W, Lee LA, Liu Y, Schepbier RW, Wen N, Zhang G, Yu X, Ning G, Wang F, Li Y, Hao L, Zhang X, Wang H. Vaccine-preventable disease control in the People’s Republic of China:1949–2016. Vaccine 2018;36:8131-8137. 9. Chen XJ, Wang HU, Li XL, Gao XY, Li MH, Fu SH, He Y, Li F, Yin QK, Xu ST, Wu D, Li YX, Yin ZD, Yang G, Liang GD. Japanese Encephalitis in China in the Period of 1950–2018: From Discovery to Control. Biomed Environ Sci, 2021; 34(3): 175-183. 10. Hsu L-C, Chen Y-U, Hsu F-K, Huang J-H, Chang C-M, Chou P, Lin I-F, Chang F-Y. The Incidence of Japanese Encephalitis in Taiwan—A Population-Based Study. PLoS Negl Trop Dis 2014;8(7)e3030.. 11. Heffelfinger JD, Li X, Batmunkh N, Grabovac G, Dioditsa S, Lyanage JB, Pattamadilok S, Bahl S, Vannice KS, Hyde TB, Chu SY, Fox KK, Hills SL, Marfin AA. Japanese Encephalitis Surveillance and Immunization — Asia and Western Pacific Regions, 2016. MMWR Morb Mortal Wkly Rep 2017;66:579–583. DOI: <http://dx.doi.org/10.15585/mmwr.mm6622a3> 12. Kono R, Kim KH. Comparative epidemiological features of Japanese encephalitis in the Republic of Korea, China (Taiwan) and Japan. Bull World Health Organ 1969;40:263-277. 13. Choe YJ, Jee Y, Takashima Y, Lee JK. Japanese encephalitis in the Western Pacific Region: Implication from the Republic of Korea. Vaccine 2020; <https://doi.org/10.1016/j.vaccine.2020.02.061>. 14. Sohn YM. Japanese encephalitis immunization in South Korea: Past, present, and future. Emerg Infect Dis 2000;6(1):17-24. 15. Sunwoo J-S, Jung K-H, Lee S-T, Lee Sk, Chu K. Reemergence of Japanese encephalitis in South Korea, 2010-2015. Emerg Infect Dis 2016;22(10):1841-3.   Abbreviations:  CD-JEV: live, attenuated SA14-14-2 JE vaccine  ELISA: enzyme-linked immunosorbent assay  IgM: Immunoglobulin M  IMOJEV: live, attenuated chimeric JE vaccine  MOH: Ministry of Health  RI: routine immunization  SIA: supplementary immunization activity | | | | | | | |
