# Supplementary material for: Impact of vaccination against Japanese encephalitis in endemic countries
Source: PLoS Negl Trop Dis. 2024 Sep 3;18(9):e0012390. doi: 10.1371/journal.pntd.0012390 (PMC11398676; doi:10.1371/journal.pntd.0012390)
Supplement: S1 Raw Data — (PDF) [file pntd.0012390.s003.pdf]

JE & AES Cases

Country: Cambodia

AES	2006	2007	2008	2009	2010	2011	2012	2013	2014	2015	2016	2017	2018
Population under 15 years		5,252,951	5,252,951	5,218,225	5,363,727	5,363,727	4,523,265	4,504,067	4,498,322	4,412,610	4,139,669	4,361,607	4,563,103
Total # AES* Cases		295	371	193	122	199	393	332	282	248	169	225	216
# AES* cases in children (under 15 years)		295	371	193	122	199	393	332	282	248	169	225	216
Total # confirmed[1] JE cases		45	36	23	41	50	69	53	60	48	10	5	11
# confirmed JE cases in children (under 15 years)		45	36	23	41	50	69	53	60	48	10	5	11

AES = acute encephalitis syndrome which is defined by WHO as a person of any age at any time of year with the acute onset of fever and at least one of the following: 1) a change in mental status (including symptoms such as confusion, disorientation, coma or inability to talk); OR 2) new onset of seizures (excluding simple febrile seizures).

* If your country uses another definition for suspected JE, please give it here: Cambodia is using ME (Meningo-Encephalitis)

A *confirmed* JE case according to the WHO's criteria for laboratory-confirmed JE:
1. Presence of IgM antibodies specific to JE virus in a single sample of cerebrospinal fluid (CSF) or serum as detected by an IgM-capture ELISA specifically for JE virus; or
2. Detection of JE-virus antigens in tissue by immunohistochemistry; or
3. Detection of JE-virus genome in serum, plasma, blood, CSF or tissue by reverse transcriptase polymerase chain reaction (PCR) or an equally sensitive and specific nucleic acid amplification test; or
4. Isolation of JE virus in serum, plasma, blood, CSF or tissue; or
5. Detection of a fourfold or greater rise in antibodies specific to JE virus as measured by haemagglutination inhibition (HI) or plaque reduction neutralization assay (PRNT) in serum collected during the acute and convalescent phase of illness. The two specimens should be collected at least 14 days apart and should be done in parallel with other confirmatory tests to eliminate the possibility of cross-reactivity with other flaviviruses such as West Nile and dengue.

2007-2015	5,252,951	5,252,951	5,218,225	5,363,727	5,363,727	4,523,265	4,504,067	4,498,322	4,412,610	44,389,845
	4,139,669	4,361,607	4,563,103							

JE Vaccinaiton Campaigns

Country: Cambodia

JE Campaigns	2006	2007	2008	2009	2010	2011	2012	2013	2014	2015	2016	2017	2018
Target population (under 15 years)*								**41,013**			**4,093,431**		
Total # of JE doses received in country for campaigns								**330,000**			**4,551,050**		
Total # children under 15 years who received at least 1 dose of JE vaccine								**39373 (96%)**			**4171429 (102%)**		
Campaign status (national/sub-national/none)								Sub-National			National		
If campaigns were subnational, please list regions where JE campaigns were conducted:								Battambang province					
Type of JE vaccine used (1)**								Live-attenuated (CD-JEV/SA 14-14-2)			Live-attenuated (CD-JEV/SA 14-14-2)		
Total # doses of JE vaccine (1) used								393373			4171429		
Type of JE vaccine used (2)**													
Total # doses of JE vaccine (2) used													
Type of JE vaccine used (3)**													
Total # doses of JE vaccine (3) used													

***"Target Population (under 15 years)"** refers to the total number of children under 15 years of age who are part of the campaign catchment area(s), regardless if they were vaccinated against JE or not.

****"Type of JE vaccine used (1)-(3)"** There are three data fields for countries that use more than one type of JE vaccine. Use the drop down list to select. If your country only uses one type of JE vaccine, please leave the other data fields blank.

JE Routine immunization

Country: Cambodia

Routine Immunization	2006	2007	2008	2009	2010	2011	2012	2013	2014	2015	2016	2017	2018
Targeted age for routine immunization (RI)				< 2 years	< 2 years	< 2 years	< 2 years			< 1 years	< 1 years	< 1 years	< 1 year
Total target population*				47,752	47,752	47,752	47,752			114,501	360,971	360,278	360,033
Total # of JE doses received in country for RI				52,500	267,000	187,000	187,000			260,000		410,000	1,063,500
Total # children who received at least 1 dose of JE vaccine				43,590	66,639	30,083	32,204			146,385	262,223	48,842	328,176
RI status (national/sub-national/none)				Sub-National	Sub-National	Sub-National	Sub-National			Sub-National	National	National	National
If RI is sub-national, please list regions where JE vaccination is part of RI:				Kampong Cham, Takeo and Svay Rieng provinces	Kampong Cham, Takeo and Svay Rieng provinces	Kampong Cham, Takeo and Svay Rieng provinces	Kampong Cham, Takeo and Svay Rieng provinces	Stock out of vaccine for 6 proces and SIA in Battambang province	Stock ou of Vaccine	Kampong Cham, Tbong Khmum, Takeo, Svay Rieng, Battambang and Bantheay Mien Chey provinces	Nation -wide SIA and Reminding vaccine from SIA and introduct nation-wide after SIA	Stock out of Vccine from January to October	
Type of JE vaccine used (1)**				Live-attenuated	Live-attenuate	Live-attenuate	Live-attenuated (CD-JEV/SA 14-14-2)			Live-attenuated (CD-JEV/SA 14-14-2)			
# of doses in the primary series for JE vaccine (1)				1	1	1	1			1	1	1	1
Total # doses of JE vaccine (1) used				43,590	66,639	30,083	32,204			146,385	262,223	48,842	328,176
Booster dose for JE vaccine (1)? (Yes/No)													
Type of JE vaccine used (2)**													
# of doses in the primary series for JE vaccine (2)													
Total # doses of JE vaccine (2) used													
Booster dose for JE vaccine (2)? (Yes/No)													
Type of JE vaccine used (3)**													
# of doses in the primary series for JE vaccine (3)													
Total # doses of JE vaccine (3) used													
Booster dose for JE vaccine (3)? (Yes/No)													

*"**Total target population**" refers to the total number of children eligible for JE vaccination as part of RI, regardless if they were vaccinated against JE or not.

"Type of JE vaccine used (1)-(3)**" There are three data fields for countries that use more than one type of JE vaccine. Use the drop down list to select. If your country only uses one type of JE vaccine, please leave the other data fields blank.

** JE lab confirmed under 15 years is extrapolated for 2011-19 based on average proportion of under 15 year cases from 2006-10 (76%)

Country: India

11-15 States up to 118 districts 2006-10 17-23 States up to 272+ dis stricts 2011-19

*Midpoint value of 2001 and 2011 census <15 years used for 2006-10. 2001 census # used for 2011-19

AES	2006	2007	2008	2009	2010	2011	2012	2013	2014	2015	2016	2017	2018	2019
Population under 15 years	*10018022(	#########	#########	#########	#########	103,863,253	103,863,253	103,863,253	103,863,253	103,863,253	103,863,253	103,863,253	103,863,253	103,863,253
Total # AES* Cases	3808	4644	4756	5668	4552	8249	8344	7825	10867	9854	11651	13672	11388	16049
# AES* cases in children (under 15 years)														
Total # confirmed[1] JE cases	214	471	646	741	563	1214	745	1086	1661	1730	1676	2181	1678	2656
# confirmed JE cases in children (under 15 years)	214	395	528	544	332	**923	566	825	1262	1315	1274	1658	1275	2019

AES = acute encephalitis syndrome which is defined by WHO as a person of any age at any time of year with the acute onset of fever and at least one of the following: 1) a change in mental status (including symptoms such as confusion, disorientation, coma or inability to talk); OR 2) new onset of seizures (excluding simple febrile seizures).

* If your country uses another definition for suspected JE, please give it here:

A *confirmed* JE case according to the WHO's criteria for laboratory-confirmed JE:

1. Presence of IgM antibodies specific to JE virus in a single sample of cerebrospinal fluid (CSF) or serum as detected by an IgM-capture ELISA specifically for JE virus; or
2. Detection of JE-virus antigens in tissue by immunohistochemistry; or
3. Detection of JE-virus genome in serum, plasma, blood, CSF or tissue by reverse transcriptase polymerase chain reaction (PCR) or an equally sensitive and specific nucleic acid amplification test; or
4. Isolation of JE virus in serum, plasma, blood, CSF or tissue; or
5. Detection of a fourfold or greater rise in antibodies specific to JE virus as measured by haemagglutination inhibition (HI) or plaque reduction neutralization assay (PRNT) in serum collected during the acute and convalescent phase of illness. The two specimens should be collected at least 14 days apart and should be done in parallel with other confirmatory tests to eliminate the possibility of cross-reactivity with other flaviviruses such as West Nile and dengue.

Country: India

JE Campaigns	2006	2007	2008	2009	2010	2011	2012	2013	2014	2015	2016	2017	2018	2019
Target population (under 15 years)*	11477194	21371482	20040260	26748158	9451232			22168436	7095256	21098077		12907950		2389891
Total # of JE doses received in country for campaigns	NA	NA	NA	NA	NA			NA	NA	NA		NA		NA
Total # children under 15 years who received at least 1 dose of JE vaccine	9308698%	18431422%	16944494	16394988	7740725			19306946	6400347	9265215		7214666		1975828
If campaigns were subnational, please list regions where JE campaigns were conducted:	11 dist- 4 State	27 dist-9 State	22 dist-8 State	29 dist-11 State	18 dist-8 State			29dist-7 States	14 dist-& 7 Stat	38 dist- 9 State		28dist-3 Sta		8dist-3 States
Campaign status (national/sub-national/none)	Sub-National	Sub-National	Sub-National	Sub-National	Sub-National			Sub-National	Sub-National	Sub-National		Sub-National		Sub-National
If campaigns were subnational, please list regions where JE campaigns were conducted:														
Type of JE vaccine used (1)**	Live-attenuated	Live-attenuated	Live-attenuated	Live-attenuated	Live-attenuated (CD-JEV/SA 14-14-2)			Live-attenuated	Live-attenuated	Live-attenuated (CD-JEV/SA 14-		Live-attenu	Live-attenuated (CD-JEV/SA 14-14-2)	
Total # doses of JE vaccine (1) used	9308698%	18431422%	16944494	16394988	7740725			19306946	6400347	9265215		7214666		1975828
Type of JE vaccine used (2)**														
Total # doses of JE vaccine (2) used														
Type of JE vaccine used (3)**														
Total # doses of JE vaccine (3) used														

"Target Population (under 15 years)" refers to the total number of children under 15 years of age who are part of the campaign catchment area(s), regardless if they were vaccinated against JE or not.

"Type of JE vaccine used (1)-(3)" There are three data fields for countries that use more than one type of JE vaccine. Use the drop down list to select. If your country only uses one type of JE vaccine, please leave the other data fields blank.

*States brought in progressively more districts over time so e.g. target for 11 States is different from one year to next

India

** 2017 vaccine #s based on April-Nov and extrapolated to full year for missing Dec 2017-Mar2018

*** These are SA 14 14 2 boosts and not 2nd doses. Nowhere else to show this substantial data

Routine Immunization	2006	2007	2008	2009	2010	2011	2012	2013	2014	2015	2016	2017	2018	2019
Targeted age for routine immunization (RI)		9-24 months	9-24 months	9-24 months	9-24 months	9-24 months	9-24 months	9-24 months	9-24 months	9-24 months	9-24 months	9-24 montl	9-24 montl	9-24 months
Total target population*		754184	1218922	4320530	7704287	82748556	4716636	8721432	9506505	10072110	10104171	9956911	10684383	10580763
Total # of JE doses received in country for RI														
Total # children who received at least 1 dose of JE vaccine		61934	116817	2245431	1614020	3759838	1692470	942014	4840423	6789137	8047084	**8493022	7711197	9682000
RI status (national/sub-national/none)		Sub-National	Sub-National	Sub-National	Sub-National	Sub-National	Sub-National	Sub-National	Sub-National	Sub-National	Sub-National	Sub-Natior	Sub-Natior	Sub-National
If RI is sub-national, please list regions where JE vaccination is part of RI:		AS, TN,UP/35	7/35 States*	11/35 States*	11/35 States*	14/35 States*	15/35 States*	147 dist-16 Sta	155 dist-16 Stat	204 dist-21 Sta	207 dist-21Sta	207dist-21	231dist-21States	
Type of JE vaccine used (1)**		Live-attenuated	Live-attenuated (CD-JEV/SA 14-		Live-attenuated	Live-attenuated	Live-attenuated	Live-attenuated	Live-attenuated	Live-attenuated	Live-attenuated	Live-attenu	Live-attenuated (CD-JEV/SA 14-14-2)	
# of doses in the primary series for JE vaccine (1)		1	1	1	1	1	1	1	1	1	1	1	1	
Total # doses of JE vaccine (1) used		61984	116817	2245431	1614020	3759838	1692470	4459037	9781972	13160220	15560317	17035270	estimate only available	
Booster dose for JE vaccine (1)? (Yes/No)		No	No	No	No	No	No	Yes	Yes	Yes	Yes	Yes	Yes	Yes
Type of JE vaccine used (2)**								Live-attenuated	Live-attenuated	Live-attenuated	Live-attenuated	Live-attenuated (CD-JEV/SA 14-14-2)		
# of doses in the primary series for JE vaccine (2)								1	1	1	1	1	1	1
Total # doses of JE vaccine (2) used								3517023	4941549	6371083	7513233	**8542248	estimate only available	
Booster dose for JE vaccine (2)? (Yes/No)														
Type of JE vaccine used (3)**														
# of doses in the primary series for JE vaccine (3)														
Total # doses of JE vaccine (3) used														
Booster dose for JE vaccine (3)? (Yes/No)														

***"Total target population"** refers to the total number of children eligible for JE vaccination as part of RI, regardless if they were vaccinated against JE or not.

****"Type of JE vaccine used (1)-(3)"** There are three data fields for countries that use more than one type of JE vaccine. Use the drop down list to select. If your country only uses one type of JE vaccine, please leave the other data fields blank.

INDIA Utter Pradesh Data							INDIA Ut
	tp6	0.33tp6	%vac (V6)	#vac	tp7	0.33tp7	v7
**Name of District**	**Projected population 2006**	**<15 in 2006**	**Campaign cov**	#Vacc in 2006	**Projected population 2007**	**<15**	**Coverage 2007**
Allahabad	5618190.78	1854002.96	0.00	0.00	5764263.74	1902207.03	0.00
Ambedkar Nagar	2280367.27	752521.20	0.00	0.00	2335096.09	770581.71	0.00
Amethi	0.00	0.00	0.00	0.00			
Azamgarh	4491823.97	1482301.91	0.00	0.00	4608611.39	1520841.76	0.00
Bahraich	2750595.08	907696.38	0.00	0.00	2830362.33	934019.57	100.00
Ballia	3053810.99	1007757.63	0.00	0.00	3117941.02	1028920.54	0.00
Balrampur	1887410.89	622845.59	0.00	0.00	1930821.34	637171.04	0.00
Barabanki	3039483.38	1003029.52	0.00	0.00	3118509.95	1029108.28	99.04
Bareilly	4091461.18	1350182.19	0.00	0.00	4201930.63	1386637.11	0.00
Basti	2306739.74	761224.11	0.00	0.00	2357488.01	777971.04	96.89
C S M Nagar	0.00	0.00	0.00	0.00			0.00
Deoria	3089169.83	1019426.04	99.86	1017968.39	3166399.08	1044911.70	0.00
Faizabad	2339326.15	771977.63	0.00	0.00	2393130.65	789733.11	0.00
Fatehpur	2558345.52	844254.02	0.00	0.00	2612070.77	861983.35	0.00
Ghazipur	3466907.28	1144079.40	0.00	0.00	3557046.87	1173825.47	0.00
Gonda	3129196.79	1032634.94	0.00	0.00	3207426.71	1058450.81	100.00
**Gorakhpur**	**4240449.78**	**1399348.43**	**97.00**	**1357367.97**	**4337980.12**	**1431533.44**	**0.00**
Hardoi	3806507.07	1256147.33	0.00	0.00	3894056.73	1285038.72	0.00
Jalaun		0.00	0.00	0.00			
Jaunpur	4339606.93	1432070.29	0.00	0.00	4430738.67	1462143.76	0.00
Kanpur Dehat	1757494.73	579973.26	0.00	0.00	1794402.12	592152.70	
Kanpur Nagar	4727037.75	1559922.46	0.00	0.00	4854667.76	1602040.36	0.00
Kushinagar	3320120.15	1095639.65	99.00	1084683.25	3413083.51	1126317.56	0.00
Lakhimpur Kheri	3745993.83	1236177.96	100.00	1236177.96	3865865.63	1275735.66	0.00
Lucknow	4330285.22	1428994.12	0.00	0.00	4473184.64	1476150.93	0.00
Maharajganj	2500022.99	825007.59	100.00	825007.59	2572523.65	848932.81	0.00
Mau	2112798.98	697223.66	0.00	0.00	2169844.55	716048.70	96.05
Muzafarnagar	3987883.43	1316001.53	0.00	0.00	4083592.63	1347585.57	0.00
Pilibhit	1872485.38	617920.18	0.00	0.00	1924914.97	635221.94	
Prabudh Nagar		0.00	0.00	0.00			
Pratapgarh	3055541.24	1008328.61	0.00	0.00	3125818.69	1031520.17	0.00
Rae Bareli	3218054.92	1061958.12	0.00	0.00	3292070.18	1086383.16	97.18
Saharanpur	3191106.74	1053065.22	0.00	0.00	3264502.20	1077285.73	0.00
Sant Kabir Nagar	1596028.43	526689.38	94.35	496931.43	1632737.08	538803.24	0.00
Shahjahanpur	2926937.41	965889.34	0.00	0.00	3008891.65	992934.25	0.00
**Shamli**		0.00	0.00	0.00			
Shrawasti	1342914.15	443161.67	0.00	0.00	1379172.83	455127.03	98.37
Siddharthnagar	2317759.65	764860.68	100.00	764860.68	2378021.40	784747.06	97.00
Sitapur	4111747.85	1356876.79	0.00	0.00	4218653.30	1392155.59	94.71
Sultanpur	3592663.29	1185578.88	0.00	0.00	0.00	1214032.78	0.00
Unnao	3010833.64	993575.10	0.00	0.00	3077071.98	1015433.75	0.00

INDIA Utter Pradesh Data							
	**Projected population 2008**	**37358343.78**	**Coverage 2008**	#VALUE!	0.00	38303688.00	25.12
	5914134.59	1951664.42	0.00	0.00		0.00	**2007.00**
	2391138.39	789075.67	0.00	0.00		0.00	
		0.00		0.00		0.00	
	4728435.29	1560383.65	100.00	1560383.65		0.00	
	2912442.84	961106.14	0.00	0.00		0.00	
	3183417.78	1050527.87	100.00	1050527.87		0.00	
	1975230.24	651825.98	0.00	0.00		0.00	
	3199591.21	1055865.10	0.00	0.00		0.00	
	4315382.76	1424076.31	105.00	1495280.13		0.00	
	2409352.75	795086.41	0.00	0.00		0.00	
		0.00	0.00	0.00		0.00	
	3245559.05	1071034.49	0.00	0.00		0.00	
	2448172.65	807896.98	99.57	804423.02		0.00	
	2666924.26	880085.01	0.00	0.00		0.00	
	3649530.09	1204344.93	0.00	0.00		0.00	
	3287612.38	1084912.08	0.00	0.00		0.00	
	**4437753.66**	**1464458.71**	**0.00**	0.00		0.00	
	3983620.04	1314594.61	100.00	1314594.61		0.00	
		0.00		0.00		0.00	
	4523784.18	1492848.78	0.00	0.00		0.00	
	1832084.57	604587.91		0.00		0.00	
	4985743.79	1645295.45	0.00	0.00		0.00	
	3508649.85	1157854.45	0.00	0.00		0.00	
	3989573.33	1316559.20	0.00	0.00		0.00	
	4620799.73	1524863.91	98.21	1497568.85		0.00	
	2647126.84	873551.86	0.00	0.00		0.00	
	2228430.36	735382.02	0.00	0.00		0.00	
	4181598.85	1379927.62	100.60	1388207.19		0.00	
	1978812.59	653008.16		0.00		0.00	
		0.00		0.00		0.00	
	3197712.52	1055245.13	0.00	0.00		0.00	
	3367787.79	1111369.97	0.00	0.00		0.00	
	3339585.75	1102063.30	0.00	0.00		0.00	
	1670290.03	551195.71	0.00	0.00		0.00	
	3093140.62	1020736.40	0.00	0.00		0.00	
		0.00		0.00		0.00	
	1416410.50	467415.46	0.00	0.00		0.00	
	2439849.96	805150.49	83.39	671414.53		0.00	
	4328338.28	1428351.63	0.00	0.00		0.00	
	3767180.50	1243169.56	99.71	1239564.37		0.00	
	3144767.56	1037773.30	0.00	0.00		0.00	
				0.00		0.00	
						0.00	
						0.00	
						0.00	

						0.00	
						0.00	
						0.00	
						0.00	
						0.00	

ter Pradesh Data							INDIA Utter Pradesh Data	
**#Vac 2007**	**0.02*0.33tp6**	**0.33tp6(0.08)**	**0.33tp6-carry forward**	**v6/100*carry forward**	**v7/100*Newborn**	**#vac**	**%Vac in 2007**	
**#vacc in 2007**	**#Lost**	**Carry forward**	**Newborns**			**Coverage**		
0.00	37080.06	148320.24	1705682.72	0.00	0.00	0.00	0.00	
0.00	15050.42	60201.70	692319.50	0.00	0.00	0.00	0.00	
0.00	0.00	0.00	0.00	0.00	0.00	0.00	#DIV/0!	
0.00	29646.04	118584.15	1363717.76	0.00	0.00	0.00	0.00	
934019.57	18153.93	72615.71	835080.67	0.00	835080.67	835080.67	89.41	
0.00	20155.15	80620.61	927137.02	0.00	0.00	0.00	0.00	
0.00	12456.91	49827.65	573017.95	0.00	0.00	0.00	0.00	
1019228.84	20060.59	80242.36	922787.15	0.00	913928.40	913928.40	88.81	
0.00	27003.64	108014.58	1242167.61	0.00	0.00	0.00	0.00	
753776.14	15224.48	60897.93	700326.18	0.00	678546.04	678546.04	87.22	
0.00	0.00	0.00	0.00	0.00	0.00	0.00	#DIV/0!	
0.00	20388.52	81554.08	937871.96	81437.47	0.00	81437.47	7.79	
0.00	15439.55	61758.21	710219.42	0.00	0.00	0.00	0.00	
0.00	16885.08	67540.32	776713.70	0.00	0.00	0.00	0.00	
0.00	22881.59	91526.35	1052553.05	0.00	0.00	0.00	0.00	
1058450.81	20652.70	82610.80	950024.15	0.00	950024.15	950024.15	89.76	
**0.00**	**27986.97**	**111947.87**	1287400.55	108589.44	0.00	108589.44	7.59	
0.00	25122.95	100491.79	1155655.55	0.00	0.00	0.00	0.00	
0.00	0.00	0.00	0.00	0.00	0.00	0.00	#DIV/0!	
0.00	28641.41	114565.62	1317504.66	0.00	0.00	0.00	0.00	
0.00	11599.47	46397.86	533575.40	0.00	0.00	0.00	0.00	
0.00	31198.45	124793.80	1435128.66	0.00	0.00	0.00	0.00	
0.00	21912.79	87651.17	1007988.48	86774.66	0.00	86774.66	7.70	
0.00	24723.56	98894.24	1137283.73	98894.24	0.00	98894.24	7.75	
0.00	28579.88	114319.53	1314674.59	0.00	0.00	0.00	0.00	
0.00	16500.15	66000.61	759006.98	66000.61	0.00	66000.61	7.77	
687764.78	13944.47	55777.89	641445.77	0.00	616108.66	616108.66	86.04	
0.00	26320.03	105280.12	1210721.41	0.00	0.00	0.00	0.00	
0.00	12358.40	49433.61	568486.56	0.00	0.00	0.00	0.00	
0.00	0.00	0.00	0.00	0.00	0.00	0.00	#DIV/0!	
0.00	20166.57	80666.29	927662.32	0.00	0.00	0.00	0.00	
1055747.15	21239.16	84956.65	977001.47	0.00	949450.03	949450.03	87.40	
0.00	21061.30	84245.22	968820.01	0.00	0.00	0.00	0.00	
0.00	10533.79	42135.15	484554.23	39754.51	0.00	39754.51	7.38	
0.00	19317.79	77271.15	888618.20	0.00	0.00	0.00	0.00	
0.00	0.00	0.00	0.00	0.00	0.00	0.00	#DIV/0!	
447708.46	8863.23	35452.93	407708.74	0.00	401063.08	401063.08	88.12	
761204.65	15297.21	61188.85	703671.83	61188.85	682561.67	743750.53	94.78	
1318510.56	27137.54	108550.14	1248326.65	0.00	1182290.17	1182290.17	84.93	
0.00	23711.58	94846.31	1090732.57	0.00	0.00	0.00	0.00	
0.00	19871.50	79486.01	914089.09	0.00	0.00	0.00	0.00	

INDIA Utter Pradesh Data								
9622324.18	747166.88	2988667.50	34369676.28	#VALUE!	8634055.48	#VALUE!	#VALUE!	
0.00			1951664.42	0.00	39169904.83	39169904.83	#DIV/0!	
				0.00	0.00	0.00	#DIV/0!	
				0.00	0.00	0.00	#DIV/0!	
				0.00	0.00	0.00	#DIV/0!	
						0.00	#DIV/0!	
						0.00	#DIV/0!	
						0.00	#DIV/0!	
						0.00		
						0.00		
						0.00		
						0.00		
						0.00		
						0.00		
						0.00		
						0.00		
						0.00		
						0.00		
						0.00		
						0.00		
						0.00		
						0.00		
						0.00		
						0.00		
						0.00		
						0.00		
						0.00		
						0.00		
						0.00		
						0.00		

						INDIA Utter Pradesh Data			
**tp8**	**0.33tp8**	**v8**	**0.02*0.33tp7**	**0.33tp7(0.08)**	**0.33tp7-carry forward**	**v7/100*carry forward**	**v8/100*New born**	**#vac in 2008**	**%Vac in 2008**
**Projected population 2008**	**<15 in 2008**	**Coverage 2008**	**#Lost**	**Carry forward**	**Newborns**			**Coverage**	
5914134.59	1951664.42	0.00	38044.14	152176.56	1799487.85	0.00	0.00	0.00	0.00
2391138.39	789075.67	0.00	15411.63	61646.54	727429.13	0.00	0.00	0.00	0.00
	0.00		0.00	0.00	0.00	#DIV/0!	0.00	#DIV/0!	#DIV/0!
4728435.29	1560383.65	100.00	30416.84	121667.34	1438716.30	0.00	1438716.30	1438716.30	92.20
2912442.84	961106.14	0.00	18680.39	74721.57	886384.57	66806.45	0.00	66806.45	6.95
3183417.78	1050527.87	100.00	20578.41	82313.64	968214.22	0.00	968214.22	968214.22	92.16
1975230.24	651825.98	0.00	12743.42	50973.68	600852.29	0.00	0.00	0.00	0.00
3199591.21	1055865.10	0.00	20582.17	82328.66	973536.44	73114.27	0.00	73114.27	6.92
4315382.76	1424076.31	105.00	27732.74	110930.97	1313145.34	0.00	1378802.61	1378802.61	96.82
2409352.75	795086.41	0.00	15559.42	62237.68	732848.72	54283.68	0.00	54283.68	6.83
	0.00	0.00	0.00	0.00	0.00	#DIV/0!	0.00	#DIV/0!	#DIV/0!
3245559.05	1071034.49	0.00	20898.23	83592.94	987441.55	6515.00	0.00	6515.00	0.61
2448172.65	807896.98	99.57	15794.66	63178.65	744718.33	0.00	741516.04	741516.04	91.78
2666924.26	880085.01	0.00	17239.67	68958.67	811126.34	0.00	0.00	0.00	0.00
3649530.09	1204344.93	0.00	23476.51	93906.04	1110438.89	0.00	0.00	0.00	0.00
3287612.38	1084912.08	0.00	21169.02	84676.07	1000236.02	76001.93	0.00	76001.93	7.01
**4437753.66**	**1464458.71**	**0.00**	28630.67	114522.68	1349936.03	8687.16	0.00	8687.16	0.59
3983620.04	1314594.61	100.00	25700.77	102803.10	1211791.51	0.00	1211791.51	1211791.51	92.18
	0.00		0.00	0.00	0.00	#DIV/0!	0.00	#DIV/0!	#DIV/0!
4523784.18	1492848.78	0.00	29242.88	116971.50	1375877.28	0.00	0.00	0.00	0.00
1832084.57	604587.91		11843.05	47372.22	557215.69	0.00	0.00	0.00	0.00
4985743.79	1645295.45	0.00	32040.81	128163.23	1517132.22	0.00	0.00	0.00	0.00
3508649.85	1157854.45	0.00	22526.35	90105.40	1067749.05	6941.97	0.00	6941.97	0.60
3989573.33	1316559.20	0.00	25514.71	102058.85	1214500.35	7911.54	0.00	7911.54	0.60
4620799.73	1524863.91	98.21	29523.02	118092.07	1406771.84	0.00	1381590.62	1381590.62	90.60
2647126.84	873551.86	0.00	16978.66	67914.62	805637.23	5280.05	0.00	5280.05	0.60
2228430.36	735382.02	0.00	14320.97	57283.90	678098.12	49288.69	0.00	49288.69	6.70
4181598.85	1379927.62	100.60	26951.71	107806.85	1272120.78	0.00	1279753.50	1279753.50	92.74
1978812.59	653008.16		12704.44	50817.76	602190.40	0.00	0.00	0.00	0.00
	0.00		0.00	0.00	0.00	#DIV/0!	0.00	#DIV/0!	#DIV/0!
3197712.52	1055245.13	0.00	20630.40	82521.61	972723.52	0.00	0.00	0.00	0.00
3367787.79	1111369.97	0.00	21727.66	86910.65	1024459.32	75956.00	0.00	75956.00	6.83
3339585.75	1102063.30	0.00	21545.71	86182.86	1015880.44	0.00	0.00	0.00	0.00
1670290.03	551195.71	0.00	10776.06	43104.26	508091.45	3180.36	0.00	3180.36	0.58
3093140.62	1020736.40	0.00	19858.68	79434.74	941301.67	0.00	0.00	0.00	0.00
	0.00		0.00	0.00	0.00	#DIV/0!	0.00	#DIV/0!	#DIV/0!
1416410.50	467415.46	0.00	9102.54	36410.16	431005.30	32085.05	0.00	32085.05	6.86
2439849.96	805150.49	83.39	15694.94	62779.76	742370.72	59500.04	619062.52	678562.56	84.28
4328338.28	1428351.63	0.00	27843.11	111372.45	1316979.19	94583.21	0.00	94583.21	6.62
3767180.50	1243169.56	99.71	24280.66	97122.62	1146046.94	0.00	1142723.41	1142723.41	91.92
3144767.56	1037773.30	0.00	20308.68	81234.70	956538.60	0.00	0.00	0.00	0.00

INDIA Utter Pradesh Data									
	39273288.64	25.33	766073.76	3064295.04					
	0.00	**2008.00**		0.00					
	0.00			0.00					
	0.00			0.00					
	0.00			0.00					
	0.00			0.00					
	0.00			0.00					
				0.00					
				0.00					
				0.00					

INDIA Utter Pradesh Data								
**tp6**	**0.33tp6**	**v6**	**0.02*0.33tp6**	**0.33tp6(0.08)**	**0.33tp6-carry forward**	**v6/100*carry forward**	**v7/100*Newborn**	**#vac**
**Projected population 2009**	**<15 in 2009**	**Coverage 2009**	**#Lost**	**Carry forward**	**Newborns**			**Coverage**
6067902.09	2002407.69	0.00	39033.29	156133.15	1795531.26	0.00	0.00	0.00
2448525.71	808013.49	4.98	15781.51	63126.05	725949.62	0.00	36122.56	36122.56
	0.00		0.00	0.00	0.00	#DIV/0!	0.00	#DIV/0!
4851374.61	1600953.62	45.62	31207.67	124830.69	1435552.95	1323619.00	654933.64	1978552.64
2996903.68	988978.22	26.38	19222.12	76888.49	884217.65	61461.94	233231.34	294693.27
3250269.55	1072588.95	25.15	21010.56	84042.23	966485.64	890757.09	243075.95	1133833.04
2020660.53	666817.98	31.20	13036.52	52146.08	599679.90	0.00	187083.47	187083.47
3282780.58	1083317.59	43.95	21117.30	84469.21	971395.89	67265.13	426900.78	494165.91
4431898.09	1462526.37	0.00	28481.53	113926.10	1310150.21	1268498.40	0.00	1268498.40
2462358.51	812578.31	52.67	15901.73	63606.91	731479.49	49940.99	385236.04	435177.03
0.00	0.00	0.00	0.00	0.00	0.00	#DIV/0!	0.00	#DIV/0!
3326698.03	1097810.35	96.50	21420.69	85682.76	985351.73	5993.80	950851.54	956845.34
2504480.62	826478.61	19.15	16157.94	64631.76	743265.22	682194.75	142362.43	824557.19
2722929.67	898566.79	97.00	17601.70	70406.80	809678.20	0.00	785387.86	785387.86
3744417.87	1235657.90	27.23	24086.90	96347.59	1107997.34	0.00	301696.63	301696.63
3369802.69	1112034.89	97.00	21698.24	86792.97	998119.12	69921.78	968175.54	1038097.32
**4539822.00**	**1498141.26**	**69.92**	29289.17	117156.70	1347302.01	7992.18	942077.18	950069.36
4075243.30	1344830.29	32.34	26291.89	105167.57	1209427.04	1114848.19	391134.53	1505982.72
	0.00		0.00	0.00	0.00	#DIV/0!	0.00	#DIV/0!
4618783.65	1524198.60	170.43	29856.98	119427.90	1373420.88	0.00	2340752.26	2340752.26
1870558.34	617284.25		12091.76	48367.03	556220.87	0.00	0.00	0.00
5120358.88	1689718.43	0.00	32905.91	131623.64	1513671.82	0.00	0.00	0.00
3606892.05	1190274.38	34.20	23157.09	92628.36	1065226.10	6386.61	364282.66	370669.27
4117239.68	1358689.09	32.69	26331.18	105324.74	1211234.46	7278.62	395976.53	403255.15
4773286.12	1575184.42	23.20	30497.28	121989.11	1402874.80	1271063.37	325409.84	1596473.21
2723893.52	898884.86	48.52	17471.04	69884.15	803667.71	4857.64	389971.29	394828.94
2288597.98	755237.33	0.00	14707.64	58830.56	676551.46	45345.60	0.00	45345.60
4281957.22	1413045.88	33.76	27598.55	110394.21	1269533.41	1177373.22	428561.19	1605934.41
2034219.35	671292.38		13060.16	52240.65	600767.50	0.00	0.00	0.00
	0.00		0.00	0.00	0.00	#DIV/0!	0.00	#DIV/0!
3271259.91	1079515.77	41.01	21104.90	84419.61	970825.52	0.00	398138.60	398138.60
3445246.91	1136931.48	40.30	22227.40	88909.60	1022460.37	69879.52	412047.33	481926.85
3416396.22	1127410.75	22.11	22041.27	88165.06	1013898.23	0.00	224136.28	224136.28
1708706.70	563873.21	97.00	11023.91	44095.66	507100.05	2925.93	491887.05	494812.98
3179748.56	1049317.02	97.00	20414.73	81658.91	939077.49	0.00	910905.17	910905.17
	0.00		0.00	0.00	0.00	#DIV/0!	0.00	#DIV/0!
1454653.58	480035.68	29.41	9348.31	37393.24	430022.23	29518.24	126474.95	155993.19
2503286.05	826084.40	65.62	16103.01	64412.04	740738.45	624277.55	486062.31	1110339.86
4440875.08	1465488.78	44.84	28567.03	114268.13	1314083.50	87016.56	589229.40	676245.96
3857592.83	1273005.63	55.72	24863.39	99453.57	1143716.00	1051305.53	637255.09	1688560.63
3213952.45	1060604.31	24.55	20755.47	83021.86	954751.43	0.00	234396.41	234396.41

INDIA Utter Pradesh Data								
	##########	43.70	785465.77			0.00	0.00	0.00
	0.00	**2009.00**	0.00			0.00	0.00	0.00
	0.00		0.00			0.00	0.00	0.00
	0.00		0.00			0.00	0.00	0.00
	0.00		0.00			0.00	0.00	0.00
	0.00		0.00			0.00	0.00	0.00
	0.00					0.00	0.00	0.00
	0.00					0.00	0.00	0.00
	0.00					0.00	0.00	0.00
	0.00					0.00	0.00	0.00
	0.00					0.00	0.00	0.00
	0.00					0.00	0.00	0.00
	0.00					0.00	0.00	0.00
	0.00					0.00	0.00	0.00
	0.00					0.00	0.00	0.00
	0.00					0.00	0.00	0.00
	0.00					0.00	0.00	0.00
	0.00					0.00	0.00	0.00
	0.00					0.00	0.00	0.00
	0.00					0.00	0.00	0.00
	0.00					0.00	0.00	0.00
	0.00					0.00	0.00	0.00
	0.00					0.00	0.00	0.00
	0.00					0.00	0.00	0.00
	0.00					0.00	0.00	0.00
	0.00					0.00	0.00	0.00
	0.00					0.00	0.00	0.00
	0.00					0.00	0.00	0.00
	0.00					0.00	0.00	0.00
	0.00					0.00	0.00	0.00
	0.00					0.00	0.00	0.00
	0.00					0.00	0.00	0.00
	0.00					0.00	0.00	0.00
	0.00					0.00	0.00	0.00
	0.00						0.00	0.00
	0.00						0.00	0.00
	0.00						0.00	0.00
	0.00						0.00	0.00
	0.00						0.00	0.00
	0.00						0.00	0.00
							0.00	0.00
							0.00	0.00
							0.00	0.00
							0.00	
							0.00	

							0.00	
							0.00	
							0.00	
							0.00	
							0.00	
							0.00	
							0.00	

INDIA Utter Pradesh Data									
%Vac in 2009						0.02*0.33tp6	0.33tp6(0.08)	0.33tp6-carry forward	v6/100 *carry forward
		Projected population 2010	<15	Recampaign	2010.00	#Lost	Carry forward	Newborns	
0.00		6225667.55	2054470.29		3.95	40048.15	160192.62	1894277.68	0.00
0.04		2507290.33	827405.81		77.92	16160.27	64641.08	762764.73	28.90
#DIV/0!			0.00			0.00	0.00	0.00	#DIV/0!
1.24		4977510.35	1642578.41		37.88	32019.07	128076.29	1514502.12	1582.84
0.30		3083813.89	1017658.58		19.68	19779.56	79118.26	938540.33	235.75
1.06		3318525.21	1095113.32		25.35	21451.78	85807.12	1009306.20	907.07
0.28		2067135.72	682154.79		14.39	13336.36	53345.44	628809.35	149.67
0.46		3368132.87	1111483.85		25.97	21666.35	86665.41	1024818.44	395.33
0.87		4551559.34	1502014.58		47.31	29250.53	117002.11	1385012.47	1014.80
0.54		2516530.39	830455.03	99.21	91.22	16251.57	65006.26	765448.77	348.14
#DIV/0!		0.00	0.00		0.00	0.00	0.00	0.00	#DIV/0!
0.87		3409865.48	1125255.61	100.00	75.87	21956.21	87824.83	1037430.78	765.48
1.00		2562083.68	845487.61		63.30	16529.57	66118.29	779369.33	659.65
0.87		2780111.19	917436.69		107.12	17971.34	71885.34	845551.35	628.31
0.24		3841772.74	1267785.00		67.39	24713.16	98852.63	1168932.37	241.36
0.93		3454047.75	1139835.76		49.42	22240.70	88962.79	1050872.97	830.48
0.63		**4644237.90**	1532598.51	**99.56**	**76.49**	29962.83	119851.30	1412747.21	760.06
1.12		4168973.89	1375761.38		58.53	26896.61	107586.42	1268174.96	1204.79
#DIV/0!			0.00			0.00	0.00	0.00	#DIV/0!
1.54		4715778.11	1556206.78		16.96	30483.97	121935.89	1434270.89	1872.60
0.00		1909840.07	630247.22			12345.69	49382.74	580864.48	0.00
0.00		5258608.57	1735340.83		64.13	33794.37	135177.47	1600163.35	0.00
0.31		3707885.03	1223602.06	98.97	20.60	23805.49	95221.95	1128380.11	296.54
0.30		4248991.35	1402167.14		30.71	27173.78	108695.13	1293472.02	322.60
1.01		4930804.56	1627165.51		36.76	31503.69	126014.75	1501150.75	1277.18
0.44		2802886.43	924952.52	97.44	49.18	17977.70	71910.79	853041.73	315.86
0.06		2350390.12	775628.74		13.14	15104.75	60418.99	715209.75	36.28
1.14		4384724.20	1446958.99		34.08	28260.92	113043.67	1333915.31	1284.75
0.00		2091177.49	690088.57			13425.85	53703.39	636385.18	0.00
#DIV/0!			0.00			0.00	0.00	0.00	#DIV/0!
0.37		3346498.89	1104344.63		28.82	21590.32	86361.26	1017983.37	318.51
0.42		3524487.59	1163080.91		88.70	22738.63	90954.52	1072126.39	385.54
0.20		3494973.33	1153341.20		74.92	22548.22	90192.86	1063148.34	179.31
0.88		1748006.96	576842.30	96.33	34.06	11277.46	45109.86	531732.44	395.85
0.87		3268781.52	1078697.90		44.37	20986.34	83945.36	994752.54	728.72
#DIV/0!			0.00			0.00	0.00	0.00	#DIV/0!
0.32		1493929.23	492996.65		49.61	9600.71	38402.85	454593.79	124.79
1.34		2568371.49	847562.59	100.00	97.00	16521.69	66086.75	781475.84	888.27
0.46		4556337.83	1503591.48		31.92	29309.78	117239.10	1386352.38	541.00
1.33		3950175.06	1303557.77		29.78	25460.11	101840.45	1201717.32	1350.85
0.22		3284659.40	1083937.60		97.00	21212.09	84848.34	999089.26	187.52

INDIA Utter Pradesh Data								
		##########		48.10	805355.58	3221422.32	38066384.30	0.00
		0.00		**2010.00**	0.00	0.00	0.00	0.00
		0.00			0.00	0.00	0.00	0.00
		0.00			0.00	0.00	0.00	0.00
		0.00			0.00	0.00	0.00	0.00
		0.00			0.00	0.00	0.00	0.00
		0.00			0.00	0.00	0.00	0.00
		0.00			0.00	0.00	0.00	0.00
		0.00			0.00	0.00	0.00	0.00
		0.00			0.00	0.00	0.00	0.00
		0.00			0.00	0.00	0.00	0.00
		0.00			0.00	0.00	0.00	0.00
		0.00			0.00	0.00	0.00	0.00
		0.00			0.00	0.00	0.00	0.00
		0.00			0.00	0.00	0.00	0.00
		0.00			0.00	0.00	0.00	0.00
		0.00			0.00	0.00	0.00	0.00
		0.00			0.00	0.00	0.00	0.00
		0.00			0.00	0.00	0.00	0.00
		0.00			0.00	0.00	0.00	0.00
		0.00			0.00	0.00	0.00	0.00
		0.00			0.00	0.00	0.00	0.00
		0.00			0.00	0.00	0.00	0.00
		0.00			0.00	0.00	0.00	0.00
		0.00			0.00	0.00		0.00
		0.00				0.00		0.00
		0.00				0.00		0.00
		0.00				0.00		0.00
		0.00				0.00		0.00
		0.00				0.00		0.00
		0.00				0.00		
		0.00				0.00		
		0.00				0.00		
		0.00				0.00		
		0.00				0.00		
		0.00				0.00		
		0.00				0.00		
		0.00				0.00		
		0.00				0.00		
		0.00				0.00		
		0.00				0.00		
		0.00				0.00		
		0.00				0.00		
		0.00				0.00		
		0.00				0.00		
		0.00				0.00		

	0.00				0.00		
	0.00				0.00		
	0.00				0.00		
	0.00				0.00		
	0.00				0.00		
	0.00				0.00		
	0.00				0.00		

INDIA Utter Pradesh Data										
v7/100*New born	#vac	%Vac in 2010				0.02*0.33tp6	0.33tp6(0.08)	0.33tp6-carry forward	v6/100*carry forward
	Coverage		Population 2011 Census	2011.00	<15	#Lost	Carry forward	Newborns	
74820.66	74820.66	3.64	5954391.00	47.26	1964949.03	41089.41	164357.62	1890112.67	5985.65
594346.06	594374.96	71.84	2397888.00	47.04	791303.04	16548.12	66192.46	761213.34	47550.00
0.00	#DIV/0!	#DIV/0!	1867000.00		616110.00	0.00	0.00	0.00	#DIV/0!
573695.15	575277.99	35.02	4613913.00	57.89	1522591.29	32851.57	131406.27	1511172.14	46022.24
184705.59	184941.35	18.17	3487731.00	37.08	1150951.23	20353.17	81412.69	936245.90	14795.31
255880.64	256787.71	23.45	3239774.00	52.48	1069125.42	21902.27	87609.07	1007504.25	20543.02
90468.72	90618.39	13.28	2148665.00	88.27	709059.45	13643.10	54572.38	627582.41	7249.47
266153.96	266549.29	23.98	3260699.00	28.86	1076030.67	22229.68	88918.71	1022565.14	21323.94
655222.47	656237.27	43.69	4448359.00	26.66	1467958.47	30040.29	120161.17	1381853.42	52498.98
698223.59	698571.73	84.12	2464464.00	62.16	813273.12	16609.10	66436.40	764018.63	55885.74
0.00	#DIV/0!	#DIV/0!	Was part of Rae Bareilly and	33.49	0.00	0.00	0.00	0.00	#DIV/0!
787063.06	787828.54	70.01	3100946.00	49.10	1023312.18	22505.11	90020.45	1035235.16	63026.28
493308.20	493967.84	58.42	2470996.00	55.57	815428.68	16909.75	67639.01	777848.60	39517.43
905787.48	906415.79	98.80	2632733.00	66.74	868801.89	18348.73	73394.94	844041.76	72513.26
787764.22	788005.57	62.16	3620268.00	48.49	1194688.44	25355.70	101422.80	1166362.20	63040.45
519338.95	520169.43	45.64	3433919.00	61.31	1133193.27	22796.72	91186.86	1048648.90	41613.55
1525855.07	1526615.13	99.61	**4440895.00**	**42.40**	1465495.35	30651.97	122607.88	1409990.63	122129.21
742265.04	743469.83	54.04	4092845.00	58.50	1350638.85	27515.23	110060.91	1265700.47	59477.59
0.00	#DIV/0!	#DIV/0!			0.00	0.00	0.00	0.00	#DIV/0!
243261.67	245134.27	15.75	4494204.00	42.44	1483087.32	31124.14	124496.54	1431710.23	19610.74
0.00	0.00	0.00	1795092.00		592380.36	12604.94	50419.78	579827.44	0.00
1026119.18	1026119.18	59.13	4581268.00	47.21	1511818.44	34706.82	138827.27	1596513.56	82089.53
1210998.96	1211295.49	98.99	3564544.00	47.00	1176299.52	24472.04	97888.16	1125713.89	96903.64
397204.43	397527.04	28.35	4021243.00	68.82	1327010.19	28043.34	112173.37	1289993.77	31802.16
551860.70	553137.88	33.99	4589838.00	38.32	1514646.54	32543.31	130173.24	1496992.27	44251.03
897203.95	897519.81	97.03	2684703.00	46.84	885951.99	18499.05	73996.20	850956.32	71801.58
94008.83	94045.11	12.13	2205968.00	79.01	727969.44	15512.57	62050.30	713578.44	7523.61
454604.39	455889.14	31.51	4143512.00	84.16	1367358.96	28939.18	115756.72	1331202.27	36471.13
0.00	0.00	0.00	2031007.00		670232.31	13801.77	55207.09	634881.48	0.00
0.00	#DIV/0!	#DIV/0!			0.00	0.00	0.00	0.00	#DIV/0!
293384.73	293703.24	26.60	3209141.00	53.93	1059016.53	22086.89	88347.57	1015997.06	23496.26
951011.59	951397.13	81.80	3209141.00	36.98	1059016.53	23261.62	93046.47	1070034.43	76111.77
796532.50	796711.81	69.08	3466382.00	54.46	1143906.06	23066.82	92267.30	1061073.90	63736.94
553768.60	554164.45	96.07	1715183.00	96.86	566010.39	11536.85	46147.38	530694.91	44333.16
441417.05	442145.77	40.99	3006538.00	59.38	992157.54	21573.96	86295.83	992402.07	35371.66
0.00	#DIV/0!	#DIV/0!			0.00	0.00	0.00	0.00	#DIV/0!
225518.84	225643.64	45.77	1117361.00	67.86	368729.13	9859.93	39439.73	453556.91	18051.49
847562.59	848450.86	100.10	2559297.00	60.61	844568.01	16951.25	67805.01	779757.59	67876.07
442563.00	443103.99	29.47	4483992.00	51.52	1479717.36	30071.83	120287.32	1383304.16	35448.32
357819.03	359169.88	27.55	3797117.00	33.02	1253048.61	26071.16	104284.62	1199273.15	28733.59
969116.58	969304.10	89.42	3108367.00	56.89	1025761.11	21678.75	86715.01	997222.60	77544.33

INDIA Utter Pradesh Data									
18310380.68	18310380.68	44.35		53.96	40081596.72	825756.13			
0.00	0.00	#DIV/0!		**2011.00**	0.00				
0.00	0.00			9.00	0.00				
0.00					0.00				
0.00					0.00				
0.00									
0.00									
0.00									
0.00									
0.00									
0.00									
0.00									
0.00									
0.00									
0.00									
0.00									
0.00									

INDIA Utter Pradesh Data									
v7/100*New born	#vac	%Vac in 2011						0.02*0.33tp6	0.33tp6(0.08)
	Coverage				Projected Population 2012	2012.00	<15	#Lost	Carry forward
893238.70	899224.35	45.76			6073478.82	27.49	2004248.01	39298.98	157195.92
358069.76	405619.76	51.26			2457835.20	54.48	811085.62	15826.06	63304.24
0.00	#DIV/0!	#####			1911808.00		630896.64	12322.20	49288.80
874788.14	920810.38	60.48			4692349.52	0.00	1548475.34	30451.83	121807.30
347205.56	362000.87	31.45			3648166.63	4.99	1203894.99	23019.02	92076.10
528712.82	549255.83	51.37			3294850.16	68.90	1087300.55	21382.51	85530.03
553981.07	561230.54	79.15			2206678.96	46.87	728204.06	14181.19	56724.76
295148.07	316472.01	29.41			3361780.67	0.00	1109387.62	21520.61	86082.45
368407.16	420906.14	28.67			4546222.90	0.00	1500253.56	29359.17	117436.68
474895.86	530781.60	65.26			2508824.35	58.96	827912.04	16265.46	65061.85
0.00	#DIV/0!	#####				47.83	0.00	0.00	0.00
508300.17	571326.45	55.83			3144359.24	85.96	1037638.55	20466.24	81864.97
432267.78	471785.21	57.86			2515473.93	56.70	830106.40	16308.57	65234.29
563272.79	635786.05	73.18			2669591.26	55.93	880965.12	17376.04	69504.15
565616.47	628656.91	52.62			3692673.36	6.58	1218582.21	23893.77	95575.08
642970.69	684584.25	60.41			3516333.06	66.49	1160389.91	22663.87	90655.46
597789.03	719918.24	49.12			**4516390.22**	**48.17**	1490408.77	29309.91	117239.63
740443.06	799920.64	59.23			4174701.90	53.05	1377651.63	27012.78	108051.11
0.00	#DIV/0!	#####					0.00	0.00	0.00
607680.52	627291.26	42.30			4561617.06	0.00	1505333.63	29661.75	118646.99
0.00	0.00	0.00			1822018.38		601266.07	11847.61	47390.43
753777.29	835866.82	55.29			4695799.70	0.00	1549613.90	30236.37	120945.48
529121.88	626025.52	53.22			3646528.51	72.80	1203354.41	23525.99	94103.96
887813.99	919616.15	69.30			4121774.08	58.45	1360185.44	26540.20	106160.82
573720.06	617971.09	40.80			4704583.95	54.26	1552512.70	30292.93	121171.72
398585.54	470387.12	53.09			2746451.17	26.11	906328.89	17719.04	70876.16
563819.42	571343.03	78.48			2247881.39	0.00	741800.86	14559.39	58237.56
1120388.96	1156860.10	84.61			4213951.70	46.80	1390604.06	27347.18	109388.72
0.00	0.00	0.00			2071627.14		683636.96	13404.65	53618.58
0.00	#DIV/0!	#####					0.00	0.00	0.00
547947.99	571444.25	53.96			3266905.54	8.42	1078078.83	21180.33	84721.32
395673.50	471785.27	44.55			3263696.40	17.86	1077019.81	21180.33	84721.32
577911.62	641648.56	56.09			3535709.64	0.00	1166784.18	22878.12	91512.48
514021.46	558354.62	98.65			1749486.66	65.08	577330.60	11320.21	45280.83
589246.03	624617.70	62.96			3060655.68	0.63	1010016.38	19843.15	79372.60
0.00	#DIV/0!	#####					0.00	0.00	0.00
307795.90	325847.39	88.37			1111774.20	12.26	366885.48	7374.58	29498.33
472618.25	540494.32	64.00			2623279.43	51.27	865682.21	16891.36	67565.44
712715.48	748163.80	50.56			4587123.82	57.68	1513750.86	29594.35	118377.39
395983.36	424716.95	33.89			3865465.11	28.29	1275603.48	25060.97	100243.89
567323.45	644867.78	62.87			3154992.51	49.54	1041147.53	20515.22	82060.89

INDIA Utter Pradesh Data									
0.00	0.00					35.20	40914337.27	801631.93	3206527.74
	0.00					**2012.00**	0.00	0.00	
	0.00						0.00	0.00	
	0.00						0.00	0.00	
	0.00					35.20	0.00	0.00	
	0.00						0.00	0.00	
	0.00						0.00	0.00	
	0.00						0.00	0.00	
	0.00						0.00		
							0.00		
							0.00		
							0.00		
							0.00		
							0.00		
							0.00		
							0.00		
							0.00		
							0.00		
							0.00		
							0.00		
							0.00		
							0.00		
							0.00		
							0.00		
							0.00		
							0.00		
							0.00		
							0.00		
							0.00		
							0.00		
							0.00		
							0.00		
							0.00		

INDIA Utter Pradesh Data								
0.33tp6-carry forward	v6/100*carry forward	v7/100*Newborn	#vac	%Vac in 2012				0.02*0.33tp6
Newborns			Coverage		Projected Population 2013	2013.00	<15	#Lost
1807753.11	71937.95	496968.15	568906.10	28.39	6194948.40	16.46	2044332.97	40084.96
727998.80	32449.58	396593.15	429042.73	52.90	2519281.08	22.89	831362.76	16221.71
566821.20	#DIV/0!	0.00	#DIV/0!	#DIV/0!	1957691.39	0.00	646038.16	12617.93
1400783.99	73664.83	0.00	73664.83	4.76	4772119.46	6.27	1574799.42	30969.51
1058875.13	28960.07	52830.07	81790.13	6.79	3815982.29	1.68	1259274.16	24077.90
983595.39	43940.47	677660.06	721600.53	66.37	3350862.61	22.49	1105784.66	21746.01
652334.69	44898.44	305774.09	350672.54	48.16	2266259.29	22.11	747865.56	14564.08
989948.22	25317.76	0.00	25317.76	2.28	3462862.34	18.27	1142744.57	22187.75
1350521.79	33672.49	0.00	33672.49	2.24	4646239.80	36.44	1533259.13	30005.07
748211.27	42462.53	441120.66	483583.18	58.41	2553983.19	28.80	842814.45	16558.24
0.00	#DIV/0!	0.00	#DIV/0!	#DIV/0!		32.80	0.00	0.00
941447.21	45706.12	809276.73	854982.85	82.40	3188380.27	26.00	1052165.49	20752.77
750194.39	37742.82	425339.59	463082.41	55.79	2560752.46	28.20	845048.31	16602.13
799297.74	50862.88	447064.19	497927.08	56.52	2706965.54	31.58	893298.63	17619.30
1099113.36	50292.55	72302.58	122595.13	10.06	3766526.83	7.54	1242953.85	24371.64
1042537.81	54766.74	693141.20	747907.94	64.45	3600725.05	17.22	1188239.27	23207.80
1348255.72	57593.46	649390.37	706983.83	47.44	**4593168.85**	**18.19**	1515745.72	29808.18
1242587.74	63993.65	659132.47	723126.12	52.49	4258195.94	20.26	1405204.66	27553.03
0.00	#DIV/0!	0.00	#DIV/0!	#DIV/0!		0.00	0.00	0.00
1364440.33	50183.30	0.00	50183.30	3.33	4630041.32	4.22	1527913.63	30106.67
544989.93	0.00	0.00	0.00	0.00	1849348.66	0.00	610285.06	12025.32
1390872.96	66869.35	0.00	66869.35	4.32	4813194.69	11.65	1588354.25	30992.28
1082195.56	50082.04	787873.96	837956.00	69.64	3730398.67	31.17	1231031.56	24067.09
1220849.37	73569.29	713544.15	787113.44	57.87	4224818.43	18.39	1394190.08	27203.71
1393474.82	49437.69	756112.17	805549.85	51.89	4822198.55	19.00	1591325.52	31050.25
815075.83	37630.97	212810.62	250441.59	27.63	2809619.55	9.98	927174.45	18126.58
669731.88	45707.44	0.00	45707.44	6.16	2290591.14	3.27	755895.08	14836.02
1257970.24	92548.81	588725.77	681274.58	48.99	4285588.88	19.03	1414244.33	27812.08
616613.73	0.00	0.00	0.00	0.00	2119274.56	0.00	699360.61	13672.74
0.00	#DIV/0!	0.00	#DIV/0!	#DIV/0!		0.00	0.00	0.00
974295.21	45715.54	82076.37	127791.91	11.85	3325709.84	32.51	1097484.25	21561.58
974295.21	37742.82	173997.35	211740.17	19.66	3319179.24	15.13	1095329.15	21540.40
1052393.58	51331.88	0.00	51331.88	4.40	3606423.83	53.59	1190119.86	23335.68
520729.56	44668.37	338909.54	383577.91	66.44	1784476.39	38.34	588877.21	11546.61
912784.94	49969.42	5778.45	55747.87	5.52	3115747.49	14.15	1028196.67	20200.33
0.00	#DIV/0!	0.00	#DIV/0!	#DIV/0!		0.00	0.00	0.00
339230.80	26067.79	41587.04	67654.83	18.44	1106215.32	20.29	365051.06	7337.71
777002.57	43239.55	398337.09	441576.63	51.01	2688861.41	16.78	887324.27	17313.64
1361339.97	59853.10	785261.69	845114.80	55.83	4692627.66	37.10	1548567.13	30275.02
1152804.72	33977.36	326080.63	360057.99	28.23	3935043.48	14.63	1298564.35	25512.07
943700.22	51589.42	467534.07	519123.49	49.86	3202317.39	25.94	1056764.74	20822.95

INDIA Utter Pradesh Data								
36875068.98			0.00	0.00		18.11	41766985.02	
0.00						**2013.00**	0.00	
							0.00	
							0.00	
							0.00	
							0.00	
							0.00	
							0.00	
							0.00	
							0.00	
							0.00	
							0.00	
							0.00	
							0.00	
							0.00	
							0.00	
							0.00	
							0.00	
							0.00	
							0.00	
							0.00	
							0.00	
							0.00	
							0.00	
							0.00	
							0.00	
							0.00	
							0.00	
							0.00	
							0.00	
							0.00	
							0.00	

INDIA Utter Pradesh Data								
0.33tp6(0.08)	0.33tp6-carry forward	v6/100*carry forward	v7/100*Newborn	#vac	%Vac in 2013			
Carry forward	Newborns			Coverage		Projected Population 2014	Campaign /recampaign coverage	2014.00
160339.84	1843908.17	45512.49	303562.23	349074.72	17.08	6318847.36		50.61
64886.85	746198.77	34323.42	170824.56	205147.98	24.68	2563620.43		2.95
50471.73	580424.91	#DIV/0!	0.00	#DIV/0!	#DIV/0!	2004675.99		34.13
123878.03	1424597.31	5893.19	89319.84	95213.03	6.05	4853245.49		24.49
96311.60	1107583.39	6543.21	18585.23	25128.44	2.00	3991517.48	67.00	7.82
86984.04	1000316.51	57728.04	224923.46	282651.50	25.56	3407827.28		50.89
58256.32	669947.73	28053.80	148126.21	176180.01	23.56	2327448.29		36.19
88751.01	1020636.61	2025.42	186505.89	188531.32	16.50	3570211.07		60.02
120020.28	1380233.27	2693.80	502951.90	505645.70	32.98	4748457.08		71.81
66232.96	761679.07	38686.65	219391.49	258078.14	30.62	2599954.89	100.00	67.08
0.00	0.00	#DIV/0!	0.00	#DIV/0!	#DIV/0!			39.66
83011.08	954627.47	68398.63	248218.19	316616.82	30.09	3233017.60	100.00	57.77
66408.51	763697.88	37046.59	215349.42	252396.01	29.87	2606846.00		59.82
70477.21	810487.91	39834.17	255957.30	295791.46	33.11	2744863.06		57.27
97486.58	1121095.63	9807.61	84509.95	94317.56	7.59	3841857.36		37.05
92831.19	1067558.72	59832.64	183824.68	243657.32	20.51	3687142.45		10.80
119232.70	1371176.07	56558.71	249431.59	305990.29	20.19	**4671252.72**	**100.00**	**31.01**
110212.13	1267439.50	57850.09	256790.39	314640.48	22.39	4343359.86		37.05
0.00	0.00	#DIV/0!	0.00	#DIV/0!	#DIV/0!			0.00
120426.69	1384906.94	4014.66	58508.87	62523.53	4.09	4699491.94		42.42
48101.29	553164.78	0.00	0.00	0.00	0.00	1877088.89	78.05	78.05
123969.11	1425644.79	5349.55	166091.33	171440.87	10.79	4933524.56		57.21
96268.35	1107086.06	67036.48	345124.21	412160.69	33.48	3816197.84	100.00	21.45
108814.84	1251370.61	62969.08	230154.90	293123.98	21.02	4330438.89	100.00	56.02
124201.02	1428311.69	64443.99	271409.44	335853.43	21.11	4942753.51		20.02
72506.31	833822.57	20035.33	83242.29	103277.62	11.14	2874240.80	100.00	42.28
59344.07	682456.79	3656.60	22350.37	26006.97	3.44	2334112.37	100.00	48.76
111248.32	1279355.74	54501.97	243413.69	297915.65	21.07	4358443.89		59.18
54690.96	628946.00	0.00	0.00	0.00	0.00	2168017.88		0.00
0.00	0.00	#DIV/0!	0.00	#DIV/0!	#DIV/0!			0.00
86246.31	991832.52	10223.35	322431.16	332654.51	30.31	3385572.61		46.96
86161.58	990858.23	16939.21	149911.52	166850.74	15.23	3375605.28		41.16
93342.73	1073441.45	4106.55	575208.14	579314.69	48.68	3678552.31		93.24
46186.45	531144.15	30686.23	203653.28	234339.51	39.79	1820165.92	100.00	64.67
80801.31	929215.07	4459.83	131471.06	135930.89	13.22	3171830.94		42.35
0.00	0.00	#DIV/0!	0.00	#DIV/0!	#DIV/0!			49.41
29350.84	337534.65	5412.39	68498.65	73911.04	20.25	1100684.25		65.99
69254.58	796427.63	35326.13	133651.04	168977.17	19.04	2756082.95	100.00	37.80
121100.07	1392650.79	67609.18	516652.89	584262.08	37.73	4800558.10		61.25
102048.28	1173555.21	28804.64	171698.31	200502.95	15.44	4005874.26		30.43
83291.80	957855.72	41529.88	248487.57	290017.45	27.44	3250352.15		43.71

INDIA Utter Pradesh Data								
		0.00	0.00	0.00				42.41
		0.00	0.00					**2014.00**
		0.00	0.00					
		0.00	0.00					
		0.00	0.00					
		0.00	0.00					
		0.00	0.00					
		0.00	0.00					
		0.00	0.00					
		0.00	0.00					
			0.00					
			0.00					
			0.00					
			0.00					
			0.00					
			0.00					
			0.00					
			0.00					
			0.00					
			0.00					
			0.00					
			0.00					
			0.00					
			0.00					
			0.00					
			0.00					
			0.00					
			0.00					
			0.00					
			0.00					
			0.00					
			0.00					
			0.00					
			0.00					
			0.00					
			0.00					
			0.00					
			0.00					
			0.00					
			0.00					
			0.00					
			0.00					
			0.00					
			0.00					
			0.00					
			0.00					

			0.00					
			0.00					
			0.00					
			0.00					
			0.00					
			0.00					
			0.00					

INDIA Utter Pradesh Data							
	0.02*0.33tp6	0.33tp6(0.08)	0.33tp6-carry forward	v6/100*carry forward	v7/100*Newborn	#vac	%Vac in 2014
<15	#Lost	Carry forward	Newborns			Coverage	
2085219.63	40886.66	163546.64	1880786.33	27925.98	951944.21	979870.19	46.99
845994.74	16627.26	66509.02	764853.74	16411.84	22537.28	38949.12	4.60
661543.08	12920.76	51683.05	594355.11	#DIV/0!	202831.10	#DIV/0!	#DIV/0!
1601571.01	31495.99	125983.95	1448815.47	7617.04	354771.62	362388.67	22.63
1317200.77	25185.48	100741.93	1158532.22	2010.28	3251674.48	3253684.76	247.02
1124583.00	22115.69	88462.77	1017321.89	22612.12	517719.72	540331.84	48.05
768057.93	14957.31	59829.25	688036.32	14094.40	248974.35	263068.75	34.25
1178169.65	22854.89	91419.57	1051325.01	15082.51	631053.99	646136.50	54.84
1566990.84	30665.18	122660.73	1410598.40	40451.66	2599954.89	2640406.54	168.50
857985.11	16856.29	67425.16	775389.30	20646.25	2599954.89	2620601.14	305.44
0.00	0.00	0.00	0.00	#DIV/0!	0.00	#DIV/0!	#DIV/0!
1066895.81	21043.31	84173.24	967992.25	25329.35	559217.23	584546.58	54.79
860259.18	16900.97	67603.86	777444.45	20191.68	465070.25	485261.93	56.41
905804.81	17865.97	71463.89	821834.74	23663.32	470691.54	494354.86	54.58
1267812.93	24859.08	99436.31	1143517.54	7545.41	423694.97	431240.38	34.01
1216757.01	23764.79	95059.14	1093180.12	19492.59	118034.44	137527.02	11.30
1541513.40	30314.91	121259.66	1394486.06	24479.22	432430.42	456909.64	29.64
1433308.75	28104.09	112416.37	1292788.29	25171.24	479040.98	504212.22	35.18
0.00	0.00	0.00	0.00	#DIV/0!	0.00	#DIV/0!	#DIV/0!
1550832.34	30558.27	122233.09	1405680.54	5001.88	596341.77	601343.65	38.78
619439.33	12205.70	48822.80	561462.25	0.00	438221.29	438221.29	70.74
1628063.10	31767.08	127068.34	1461285.91	13715.27	835989.87	849705.14	52.19
1259345.29	24620.63	98482.52	1132549.04	32972.86	242898.64	275871.50	21.91
1429044.83	27883.80	111535.21	1282654.87	23449.92	718554.29	742004.21	51.92
1631108.66	31826.51	127306.04	1464019.48	26868.27	293169.36	320037.63	19.62
948499.46	18543.49	74173.96	853000.49	8262.21	360634.89	368897.10	38.89
770257.08	15117.90	60471.61	695423.47	2080.56	339070.68	341151.23	44.29
1438286.49	28284.89	113139.55	1301104.78	23833.25	769930.47	793763.72	55.19
715445.90	13987.21	55948.85	643411.76	0.00	0.00	0.00	0.00
0.00	0.00	0.00	0.00	#DIV/0!	0.00	#DIV/0!	#DIV/0!
1117238.96	21949.68	87798.74	1009685.51	26612.36	474136.01	500748.37	44.82
1113949.74	21906.58	87626.33	1007702.82	13348.06	414754.96	428103.02	38.43
1213922.26	23802.40	95209.59	1094910.28	46345.18	1020908.69	1067253.87	87.92
600654.75	11777.54	47110.18	541767.03	18747.16	350361.15	369108.31	61.45
1046704.21	20563.93	82255.73	945940.94	10874.47	400633.97	411508.44	39.31
0.00	0.00	0.00	0.00	#DIV/0!	0.00	#DIV/0!	#DIV/0!
363225.80	7301.02	29204.08	335846.97	5912.88	221609.73	227522.62	62.64
909507.37	17746.49	70985.94	816338.32	13518.17	308604.32	322122.49	35.42
1584184.17	30971.34	123885.37	1424681.76	46740.97	872631.58	919372.55	58.03
1321938.51	25971.29	103885.15	1194679.20	16040.24	363599.22	379639.46	28.72
1072616.21	21135.29	84541.18	972223.56	23201.40	424942.98	448144.37	41.78

INDIA Utter Pradesh Data							
42633932.13	835339.70	3341358.80			0.00		0.00
0.00					0.00		#DIV/0!
0.00					0.00		#DIV/0!
0.00					0.00		#DIV/0!
0.00					0.00		#DIV/0!
0.00					0.00		#DIV/0!
0.00					0.00		#DIV/0!
0.00					0.00		
0.00					0.00		
0.00					0.00		
0.00					0.00		
0.00					0.00		
0.00					0.00		
0.00					0.00		
0.00					0.00		
0.00					0.00		
0.00					0.00		
0.00					0.00		
0.00					0.00		
0.00					0.00		
0.00					0.00		
0.00					0.00		
0.00					0.00		
0.00					0.00		
0.00					0.00		
0.00					0.00		
0.00					0.00		
0.00					0.00		
0.00					0.00		
0.00					0.00		
0.00					0.00		
0.00					0.00		
0.00					0.00		
0.00					0.00		
0.00					0.00		
0.00					0.00		
0.00					0.00		
0.00					0.00		
0.00					0.00		
0.00					0.00		
0.00					0.00		
0.00							
0.00							
0.00							
0.00							

0.00							
0.00							
0.00							
0.00							
0.00							
0.00							
0.00							

INDIA Utter Pradesh Data							
					0.02*0.33tp6	0.33tp6(0.08)	0.33tp6-carry forward
	Projected Population 2015	Campaign cov	2015.00	<15	#Lost	Carry forward	Newborns
	6445224.31		75.95	2126924.02	41704.39	166817.57	1918402.06
	2608740.15		49.04	860884.25	16919.89	67679.58	778315.16
	2052788.21	43.79	11.38	677420.11	13230.86	52923.45	608619.63
	4935750.67		33.35	1628797.72	32031.42	128125.68	1473445.33
	4175127.28		23.86	1377792.00	26344.02	105376.06	1211824.71
	3465760.34		69.47	1143700.91	22491.66	89966.64	1034616.36
	2390289.39		53.65	788795.50	15361.16	61444.63	706613.30
	3680887.61		71.06	1214692.91	23563.39	94253.57	1083916.08
	4852923.13		83.48	1601464.63	31339.82	125359.27	1441631.57
	2646754.08		78.90	873428.84	17159.70	68638.81	789346.30
			11.10	0.00	0.00	0.00	0.00
	3278279.84		81.50	1081832.35	21337.92	85351.66	981544.14
	2653769.23		79.01	875743.85	17205.18	68820.73	791438.45
	2783291.14		76.84	918486.08	18116.10	72464.38	833340.42
	3918694.51		62.16	1293169.19	25356.26	101425.03	1166387.90
	3775633.87		40.03	1245959.18	24335.14	97340.56	1119416.45
	**4750664.02**		**68.59**	1567719.13	30830.27	123321.07	1418192.33
	4430227.05		61.99	1461974.93	28666.18	114664.70	1318644.05
			0.00	0.00	0.00	0.00	0.00
	4769984.31		78.72	1574094.82	31016.65	124066.59	1426765.75
	1905245.22		29.33	628730.92	12388.79	49555.15	569884.19
	5056862.67		97.00	1668764.68	32561.26	130245.05	1497818.06
	3903970.39		34.06	1288310.23	25186.91	100747.62	1158597.66
	4438699.86		71.28	1464770.95	28580.90	114323.59	1314721.25
	5066322.35		79.08	1671886.38	32622.17	130488.69	1500619.97
	2940348.33		74.54	970314.95	18969.99	75879.96	872619.51
	2378460.51		84.01	784891.97	15405.14	61620.57	708636.52
	4432537.44		55.99	1462737.36	28765.73	115062.92	1323223.57
	2217882.29	56.16	50.49	731901.16	14308.92	57235.67	658210.23
			0.00	0.00	0.00	0.00	0.00
	3446512.92		58.13	1137349.26	22344.78	89379.12	1027859.85
	3432990.57		54.30	1132886.89	22278.99	89115.98	1024833.76
	3752123.36		80.92	1238200.71	24278.45	97113.78	1116808.48
	1856569.24		74.24	612667.85	12013.10	48052.38	552602.37
	3228923.90		64.00	1065544.89	20934.08	83736.34	962967.87
			16.47	0.00	0.00	0.00	0.00
	1095180.83		75.02	361409.67	7264.52	29058.06	334167.74
	2824985.02		59.89	932245.06	18190.15	72760.59	836746.78
	4910970.94		67.40	1620620.41	31683.68	126734.73	1457449.44
	4077980.00		33.50	1345733.40	26438.77	105755.08	1216183.43
	3299107.44		61.80	1088705.45	21452.32	85809.30	986806.91

INDIA Utter Pradesh Data						
		56.87	43520552.59	852678.64		42633932.13
		**2015.00**				0.00
						0.00
						0.00
						0.00
						0.00
						0.00
						0.00
						0.00

INDIA Utter Pradesh Data						
v6/100*carry forward	v7/100*Newborn	#vac	%Vac in 2015			
		Coverage		Projected population 2016	2016.00	<15
78389.62	1456953.63	1535343.25	72.19	6574128.80	84.40	2169462.50
3115.93	381670.40	384786.33	44.70	2654653.97	16.35	876035.81
#DIV/0!	69233.02	#DIV/0!	#DIV/0!	2102055.13	56.10	693678.19
28991.09	491436.97	520428.06	31.95	5019658.43	53.00	1656487.28
260294.78	289182.60	549477.38	39.88	4367183.13	47.45	1441170.43
43226.55	718742.76	761969.31	66.62	3524678.26	78.77	1163143.83
21045.50	379062.94	400108.44	50.72	2454827.20	64.16	810092.98
51690.92	770242.99	821933.91	67.67	3785102.81	72.26	1249083.93
211232.52	1203480.95	1414713.47	88.34	4959687.44	79.36	1636696.86
209648.09	622822.23	832470.32	95.31	2694395.65	79.80	889150.56
#DIV/0!	0.00	#DIV/0!	#DIV/0!		0.00	0.00
46763.73	800006.36	846770.08	78.27	3324175.76	84.59	1096978.00
38820.95	625308.26	664129.21	75.84	2701537.08	82.16	891507.24
39548.39	640377.58	679925.97	74.03	2822257.22	81.69	931344.88
34499.23	724981.89	759481.12	58.73	3997068.40	80.57	1319032.57
11002.16	448120.94	459123.10	36.85	3866249.08	68.02	1275862.20
36552.77	972731.65	1009284.42	64.38	**4831425.30**	**77.26**	1594370.35
40336.98	817413.18	857750.15	58.67	4518831.59	70.95	1491214.43
#DIV/0!	0.00	#DIV/0!	#DIV/0!		0.00	0.00
48107.49	1123094.23	1171201.72	74.40	4841534.08	84.41	1597706.25
35057.70	167131.91	202189.62	32.16	1933823.90	54.43	638161.89
67976.41	1452883.51	1520859.93	91.14	5183284.24	97.00	1710483.80
22069.72	394636.38	416706.10	32.35	3993761.71	59.48	1317941.36
59360.34	937193.51	996553.85	68.03	4549667.36	70.39	1501390.23
25603.01	1186657.25	1212260.26	72.51	5192980.41	96.10	1713683.53
29511.77	650439.18	679950.95	70.08	3007976.35	83.85	992632.19
27292.10	595356.52	622648.62	79.33	2423651.25	90.38	799804.91
63501.10	740873.03	804374.13	54.99	4507890.58	46.44	1487603.89
0.00	332335.47	332335.47	45.41	2268893.58	80.03	748734.88
#DIV/0!	0.00	#DIV/0!	#DIV/0!		0.00	0.00
40059.87	597464.41	637524.28	56.05	3508550.15	68.31	1157821.55
34248.24	556437.34	590685.58	52.14	3491351.41	65.64	1152145.97
85380.31	903695.51	989075.81	79.88	3827165.82	76.98	1262964.72
29528.66	410232.03	439760.70	71.78	1893700.62	71.30	624921.21
32920.68	616338.17	649258.84	60.93	3287044.53	71.40	1084724.69
#DIV/0!	0.00	#DIV/0!	#DIV/0!		0.00	0.00
18201.81	250678.16	268879.97	74.40	1089704.92	75.41	359602.62
25769.80	501143.80	526913.60	56.52	2895609.65	61.62	955551.18
73549.80	982278.26	1055828.06	65.15	5023923.27	67.47	1657894.68
30371.16	407376.98	437748.13	32.53	4151383.64	40.09	1369956.60
35851.55	609836.85	645688.40	59.31	3348594.05	70.43	1105036.04

INDIA Utter Pradesh Data			
0.00	24244238.95	24244238.95	55.71
#DIV/0!	0.00	#DIV/0!	#DIV/0!
#DIV/0!	0.00	#DIV/0!	#DIV/0!
#DIV/0!	0.00	#DIV/0!	#DIV/0!
#DIV/0!	0.00	#DIV/0!	#DIV/0!
#DIV/0!	0.00	#DIV/0!	#DIV/0!
	0.00	0.00	#DIV/0!
	0.00	0.00	#DIV/0!
	0.00	0.00	#DIV/0!
	0.00	0.00	#DIV/0!
	0.00	0.00	#DIV/0!
	0.00	0.00	#DIV/0!
	0.00	0.00	#DIV/0!
	0.00	0.00	#DIV/0!
	0.00	0.00	
	0.00	0.00	
	0.00	0.00	
	0.00	0.00	
	0.00	0.00	
	0.00	0.00	
	0.00	0.00	
	0.00	0.00	
	0.00	0.00	
	0.00	0.00	
	0.00	0.00	
	0.00	0.00	
	0.00		
	0.00		
	0.00		
	0.00		
	0.00		
	0.00		
	0.00		
	0.00		
	0.00		
	0.00		
	0.00		
	0.00		
	0.00		
	0.00		
	0.00		
	0.00		
	0.00		
	0.00		
	0.00		
	0.00		

INDIA Utter Pradesh Data		
	63.61	44424074.24
	**2016.00**	0.00
		0.00
		0.00
		0.00
		0.00
		0.00
		0.00
		0.00
		0.00
		0.00
		0.00
		0.00
		0.00
		0.00
		0.00
		0.00
		0.00
		0.00
		0.00
		0.00
		0.00
		0.00
		0.00
		0.00
		0.00
		0.00
		0.00
		0.00
		0.00
		0.00
		0.00
		0.00
		0.00
		0.00
		0.00
		0.00
		0.00
		0.00
		0.00
		0.00

	0.00		
	0.00		
	0.00		
	0.00		
	0.00		
	0.00		
	0.00		

INDIA Utter Pradesh Data						
0.02*0.33tp6	0.33tp6(0.08)	0.33tp6-carry forward	v6/100*carry forward	v7/100*Newborn	#vac	%Vac in 2016
#Lost	Carry forward	Newborns			Coverage	
42538.48	170153.92	1956770.10	122827.46	1651539.70	1774367.16	81.79
17217.68	68870.74	792013.51	30782.91	129462.60	160245.51	18.29
13548.40	54193.61	623226.50	#DIV/0!	349649.97	#DIV/0!	#DIV/0!
32575.95	130303.82	1498493.90	41634.24	794170.79	835805.03	50.46
27555.84	110223.36	1267568.64	43958.19	601412.87	645371.06	44.78
22874.02	91496.07	1052204.84	60957.54	828820.45	889778.00	76.50
15775.91	63103.64	725691.86	32008.67	465591.81	497600.49	61.43
24293.86	97175.43	1117517.48	65754.71	807559.97	873314.69	69.92
32029.29	128117.17	1473347.46	113177.08	1169311.00	1282488.08	78.36
17468.58	69874.31	803554.54	66597.63	641266.13	707863.76	79.61
0.00	0.00	0.00	#DIV/0!	0.00	#DIV/0!	#DIV/0!
21636.65	86546.59	995285.76	67741.61	841905.14	909646.75	82.92
17514.88	70059.51	805684.34	53130.34	661947.61	715077.94	80.21
18369.72	73478.89	845007.19	54394.08	690290.70	744684.77	79.96
25863.38	103453.54	1189715.65	60758.49	958529.32	1019287.80	77.28
24919.18	99676.73	1146282.44	36729.85	779664.50	816394.35	63.99
31354.38	125417.53	1442301.60	80742.75	1114374.03	1195116.78	74.96
29239.50	116957.99	1345016.93	68620.01	954347.56	1022967.57	68.60
0.00	0.00	0.00	#DIV/0!	0.00	#DIV/0!	#DIV/0!
31481.90	125927.59	1448167.24	93696.14	1222326.22	1316022.36	82.37
12574.62	50298.47	578432.45	16175.17	314863.26	331038.43	51.87
33375.29	133501.17	1535263.51	121668.79	1489205.60	1610874.40	94.18
25766.20	103064.82	1185245.41	33336.49	705042.21	738378.70	56.03
29295.42	117181.68	1347589.28	79724.31	948626.30	1028350.61	68.49
33437.73	133750.91	1538135.47	96980.82	1478202.00	1575182.82	91.92
19406.30	77625.20	892689.75	54396.08	748499.80	802895.88	80.89
15697.84	62791.36	722100.61	49811.89	652646.20	702458.09	87.83
29254.75	117018.99	1345718.37	64349.93	624998.91	689348.84	46.34
14638.02	58552.09	673349.06	26586.84	538910.52	565497.36	75.53
0.00	0.00	0.00	#DIV/0!	0.00	#DIV/0!	#DIV/0!
22746.99	90987.94	1046361.32	51001.94	714751.59	765753.54	66.14
22657.74	90630.95	1042255.94	47254.85	684142.78	731397.63	63.48
24764.01	99056.06	1139144.65	79126.07	876921.94	956048.00	75.70
12253.36	49013.43	563654.42	35180.86	401896.66	437077.52	69.94
21310.90	85243.59	980301.30	51940.71	699946.29	751887.00	69.32
0.00	0.00	0.00	#DIV/0!	0.00	#DIV/0!	#DIV/0!
7228.19	28912.77	332496.90	21510.40	250738.40	272248.79	75.71
18644.90	74579.60	857665.45	42153.09	528491.09	570644.18	59.72
32412.41	129649.63	1490970.78	84466.25	1005892.46	1090358.71	65.77
26914.67	107658.67	1238074.73	35019.85	496338.30	531358.15	38.79
21774.11	87096.44	1001609.02	51655.07	705430.18	757085.25	68.51

INDIA Utter Pradesh Data						
870411.05	3481644.21	40038908.39	1939539.12	25469369.33	27408908.45	61.70
0.00		0.00	#DIV/0!	0.00	#DIV/0!	#DIV/0!
0.00		0.00	#DIV/0!	0.00	#DIV/0!	#DIV/0!
0.00		0.00	#DIV/0!	0.00	#DIV/0!	#DIV/0!
0.00		0.00	#DIV/0!	0.00	#DIV/0!	#DIV/0!
0.00		0.00	#DIV/0!	0.00	#DIV/0!	#DIV/0!
0.00		0.00	#DIV/0!	0.00	#DIV/0!	#DIV/0!
0.00		0.00	#DIV/0!	0.00	#DIV/0!	#DIV/0!
0.00		0.00	#DIV/0!	0.00	#DIV/0!	#DIV/0!
0.00		0.00	#DIV/0!	0.00	#DIV/0!	#DIV/0!
0.00		0.00	#DIV/0!	0.00	#DIV/0!	#DIV/0!
0.00		0.00	0.00	0.00	0.00	#DIV/0!
0.00		0.00	0.00	0.00	0.00	#DIV/0!
0.00		0.00	0.00	0.00		#DIV/0!
0.00		0.00	0.00	0.00		#DIV/0!
0.00		0.00	0.00	0.00		#DIV/0!
0.00		0.00	0.00	0.00		#DIV/0!
0.00		0.00	0.00	0.00		#DIV/0!
0.00		0.00	0.00	0.00		#DIV/0!
0.00		0.00	0.00	0.00		#DIV/0!
0.00		0.00	0.00	0.00		#DIV/0!
0.00		0.00	0.00	0.00		#DIV/0!
0.00		0.00	0.00	0.00		#DIV/0!
0.00		0.00	0.00	0.00		#DIV/0!
0.00		0.00	0.00	0.00		#DIV/0!
0.00		0.00	0.00	0.00		#DIV/0!
0.00		0.00	0.00	0.00		#DIV/0!
		0.00	0.00	0.00		#DIV/0!
		0.00	0.00	0.00		#DIV/0!
		0.00	0.00	0.00		#DIV/0!
		0.00	0.00	0.00		#DIV/0!
		0.00	0.00	0.00		#DIV/0!
		0.00	0.00	0.00		#DIV/0!
		0.00	0.00	0.00		#DIV/0!
		0.00	0.00	0.00		#DIV/0!
		0.00	0.00	0.00		#DIV/0!
		0.00	0.00	0.00		#DIV/0!
		0.00	0.00	0.00		#DIV/0!
		0.00	0.00	0.00		#DIV/0!
		0.00	0.00	0.00		#DIV/0!
		0.00	0.00	0.00		#DIV/0!
		0.00	0.00	0.00		#DIV/0!
		0.00	0.00	0.00		#DIV/0!
		0.00	0.00	0.00		#DIV/0!
		0.00	0.00	0.00		#DIV/0!
		0.00	0.00	0.00		#DIV/0!

		0.00	0.00	0.00		#DIV/0!
		0.00	0.00	0.00		#DIV/0!
		0.00	0.00	0.00		#DIV/0!
		0.00	0.00	0.00		
		0.00	0.00	0.00		
		0.00	0.00	0.00		
		0.00	0.00			

Country: Indonesia Indonesia

AES	2006	2007	2008	2009	2010	2011	2012	2013	2014	2015	2016	2017	2018
Population under 15 years										69857406	70096861	70295363	70486717
Total # AES* Cases										317	326	281	231
# AES* cases in children (under 15 years)										317	326	281	231
Total # confirmed[1] JE cases										39	43	6	6
# confirmed JE cases in children (under 15 years)										39	43	6	6

AES = acute encephalitis syndrome which is defined by WHO as a person of any age at any time of year with the acute onset of fever and at least one of the following: 1) a change in mental status (including symptoms such as confusion, disorientation, coma or inability to talk); OR 2) new onset of seizures (excluding simple febrile seizures).

* If your country uses another definition for suspected JE, please give it here:

A *confirmed* JE case according to the WHO's criteria for laboratory-confirmed JE:
1. Presence of IgM antibodies specific to JE virus in a single sample of cerebrospinal fluid (CSF) or serum as detected by an IgM-capture ELISA specifically for JE virus; or
2. Detection of JE-virus antigens in tissue by immunohistochemistry; or
3. Detection of JE-virus genome in serum, plasma, blood, CSF or tissue by reverse transcriptase polymerase chain reaction (PCR) or an equally sensitive and specific nucleic acid amplification test; or
4. Isolation of JE virus in serum, plasma, blood, CSF or tissue; or
5. Detection of a fourfold or greater rise in antibodies specific to JE virus as measured by haemagglutination inhibition (HI) or plaque reduction neutralization assay (PRNT) in serum collected during the acute and convalescent phase of illness. The two specimens should be collected at least 14 days apart and should be done in parallel with other confirmatory tests to eliminate the possibility of cross-reactivity with other flaviviruses such as West Nile and dengue.

Bali Province

AES	2006	2007	2008	2009	2010	2011	2012	2013	2014	2015	2016	2017	2018
Population under 15 years										1019312	1018906	1015140	1010769
Total # AES* Cases										207	226	133	95
# AES* cases in children (under 15 years)										207	226	133	95
Total # confirmed[1] JE cases										22	17	4	1
# confirmed JE cases in children (under 15 years)										22	17	4	1

Country: Indonesia

JE Campaigns	2006	2007	2008	2009	2010	2011	2012	2013	2014	2015	2016	2017	2018
Target population (9 months - 14 years)*													962,810
Total # of JE doses received in country for campaigns													1,194,830
Total # children under 15 years who received at least 1 dose of JE vaccine													979,953
Campaign status (national/sub-national/none)													Sub-National
If campaigns were subnational, please list regions where JE campaigns were conducted:													Bali
Type of JE vaccine used (1)**													Live-attenuated (CD-JEV/SA 14-14-2)
Total # doses of JE vaccine (1) used													1,164,275
Type of JE vaccine used (2)**													
Total # doses of JE vaccine (2) used													
Type of JE vaccine used (3)**													
Total # doses of JE vaccine (3) used													

***"Target Population (9 months - < 15 years)"** refers to the total number of children under 15 years of age who are part of the campaign catchment area(s), regardless if they were vaccinated against JE or not.
****"Type of JE vaccine used (1)-(3)"** There are three data fields for countries that use more than one type of JE vaccine. Use the drop down list to select. If your country only uses one type of JE vaccine, please leave the other data fields blank.

Country: Indonesia

Routine Immunization	2006	2007	2008	2009	2010	2011	2012	2013	2014	2015	2016	2017	2018
Targeted age for routine immunization (RI)													10 mos
Total target population*													46,624
Total # of JE doses received in country for RI													51,975
Total # children who received at least 1 dose of JE vaccine													22,522
RI status (national/sub-national/none)													Sub-National
If RI is sub-national, please list regions where JE vaccination is part of RI:													Bali
Type of JE vaccine used (1)**													Live-attenuated (CD-JEV/SA 14-14-2)
# of doses in the primary series for JE vaccine (1)													1
Total # doses of JE vaccine (1) used													22,522
Booster dose for JE vaccine (1)? (Yes/No)													No
Type of JE vaccine used (2)**													None (N/A)
# of doses in the primary series for JE vaccine (2)													
Total # doses of JE vaccine (2) used													
Booster dose for JE vaccine (2)? (Yes/No)													
Type of JE vaccine used (3)**													
# of doses in the primary series for JE vaccine (3)													
Total # doses of JE vaccine (3) used													
Booster dose for JE vaccine (3)? (Yes/No)													

***"Total target population"** refers to the total number of children eligible for JE vaccination as part of RI, regardless if they were vaccinated against JE or not.

****"Type of JE vaccine used (1)-(3)"** There are three data fields for countries that use more than one type of JE vaccine. Use the drop down list to select. If your country only uses one type of JE vaccine, please leave the other data fields blank.

Note: # children vaccinated covered the period of May - December 2018, as JE vaccine was introduced in Bali for routine program started in May 2018.

JE & AES Cases

Country: Laos

AES	2006	2007	2008	2009	2010	2011	2012	2013	2014	2015	2016	2017	2018
Population under 15 years	1,839,831	1,878,679	1,918,114	1,961,025	2,003,154	2,046,470	2,091,010	2,136,813	2,183,921	2,134,937	2,171,842	2,208,270	2,244,159
Total # AES* Cases	13	25	14	72	50	36	24	24	37	26	19	12	9
# AES* cases in children (under 15 years)	10	21	13	50	35	25	21	14	30	20	14	10	7
Total # confirmed[1] JE cases					47	24	23	6	16	31	19	9	11
# confirmed JE cases in children (under 15 years)					35	16	22	3	9	25	11	7	11

AES = acute encephalitis syndrome which is defined by WHO as a person of any age at any time of year with the acute onset of fever and at least one of the following: 1) a change in mental status (including symptoms such as confusion, disorientation, coma or inability to talk); OR 2) new onset of seizures (excluding simple febrile seizures).

* If your country uses another definition for suspected JE, please give it here:

A *confirmed* JE case according to the WHO's criteria for laboratory-confirmed JE:
1. Presence of IgM antibodies specific to JE virus in a single sample of cerebrospinal fluid (CSF) or serum as detected by an IgM-capture ELISA specifically for JE virus; or
2. Detection of JE-virus antigens in tissue by immunohistochemistry; or
3. Detection of JE-virus genome in serum, plasma, blood, CSF or tissue by reverse transcriptase polymerase chain reaction (PCR) or an equally sensitive and specific nucleic acid amplification test; or
4. Isolation of JE virus in serum, plasma, blood, CSF or tissue; or
5. Detection of a fourfold or greater rise in antibodies specific to JE virus as measured by haemagglutination inhibition (HI) or plaque reduction neutralization assay (PRNT) in serum collected during the acute and convalescent phase of illness. The two specimens should be collected at least 14 days apart and should be done in parallel with other confirmatory tests to eliminate the possibility of cross-reactivity with other flaviviruses such as West Nile and dengue.

2010-2014

2,003,154
2,046,470
2,091,010
2,136,813
2,183,921

2015-2018

2,134,937
2,171,842
2,208,270
2,244,159
8,759,209

JE vaccination campaigns

Country: Laos

JE Campaigns	2006	2007	2008	2009	2010	2011	2012	2013	2014	2015	2016	2017	2018
Target population (under 15 years)*	1,839,831	1,878,679	1,918,114	1,961,025	2,003,154	2,046,470	2,091,010	2,136,813	2,183,921	2,134,937	2,171,842	2,208,270	2,244,159
Total # of JE doses received in country for campaigns								699,950		1521000			
Total # children under 15 years who received at least 1 dose of JE vaccine								564,865		1,460,894			
Campaign status (national/sub-national/none)								Sub-National		Sub-National			
If campaigns were subnational, please list regions where JE campaigns were conducted:													
Type of JE vaccine used (1)**								Live-attenuated (CD-JEV/S		Live-attenuated (CD-JEV/SA 14-14-2)			
Total # doses of JE vaccine (1) used								564865		1460894			
Type of JE vaccine used (2)**													
Total # doses of JE vaccine (2) used													
Type of JE vaccine used (3)**													
Total # doses of JE vaccine (3) used													

***"Target Population (under 15 years)"** refers to the total number of children under 15 years of age who are part of the campaign catchment area(s), regardless if they were vaccinated against JE or not.

****"Type of JE vaccine used (1)-(3)"** There are three data fields for countries that use more than one type of JE vaccine. Use the drop down list to select. If your country only uses one type of JE vaccine, please leave the other data fields blank.

JE Routine Immunization

Country: Laos

Routine Immunization	2006	2007	2008	2009	2010	2011	2012	2013	2014	2015	2016	2017	2018
Targeted age for routine immunization (RI)	under 1	under 1	under 1	under 1	under 1	under 1	under 1	under 1	under 1	under 1	under 1	under 1	under 1
Total target population*	152,419	155,610	158,850	162,424	165,886	169,445	173,105	176,869	180,741	156,235	155,578	154,987	154,395
Total # of JE doses received in country for RI									233500	233500		100000	627000
Total # children who received at least 1 dose of JE vaccine											80,594	68,214	108,724
RI status (national/sub-national/none)											National	National	National
If RI is sub-national, please list regions where JE vaccination is part of RI:													
Type of JE vaccine used (1)**											Live-attenu	Live-attenu	Live-attenuated (CD-JEV/SA 14-14-2)
# of doses in the primary series for JE vaccine (1)											1	1	1
Total # doses of JE vaccine (1) used											80594	68214	108724
Booster dose for JE vaccine (1)? (Yes/No)													
Type of JE vaccine used (2)**													
# of doses in the primary series for JE vaccine (2)													
Total # doses of JE vaccine (2) used													
Booster dose for JE vaccine (2)? (Yes/No)													
Type of JE vaccine used (3)**													
# of doses in the primary series for JE vaccine (3)													
Total # doses of JE vaccine (3) used													
Booster dose for JE vaccine (3)? (Yes/No)													

***"Total target population"** refers to the total number of children eligible for JE vaccination as part of RI, regardless if they were vaccinated against JE or not.

****"Type of JE vaccine used (1)-(3)"** There are three data fields for countries that use more than one type of JE vaccine. Use the drop down list to select. If your country only uses one type of JE vaccine, please leave the other data fields blank.

Myanmar

AES	2006	2007	2008	2009	2010	2011	2012	2013	2014	2015	2016	2017	2018	2019	2020
Population under 15 years												13.6 million	13271916	13271916	13181445
Total # AES* Cases	NA	NA	NA	NA	NA	NA	175	226	152	645	1911	2089	2063	2038	860
# AES* cases in children (under 15 years)															
Total # confirmed[1] JE cases							14	17	24	151	383	323	126	115	75
# confirmed JE cases in children (under 15 years)							6	12	24	53	342	232	96	84	66

AES = acute encephalitis syndrome which is defined by WHO as a person of any age at any time of year with the acute onset of fever and at least one of the following: 1) a change in mental status (including symptoms such as confusion, disorientation, coma or inability to talk); OR 2) new onset of seizures (excluding simple febrile seizures).

* If your country uses another definition for suspected JE, please give it here:

A *confirmed* JE case according to the WHO's criteria for laboratory-confirmed JE:
1. Presence of IgM antibodies specific to JE virus in a single sample of cerebrospinal fluid (CSF) or serum as detected by an IgM-capture ELISA specifically for JE virus; or
2. Detection of JE-virus antigens in tissue by immunohistochemistry; or
3. Detection of JE-virus genome in serum, plasma, blood, CSF or tissue by reverse transcriptase polymerase chain reaction (PCR) or an equally sensitive and specific nucleic acid amplification test; or
4. Isolation of JE virus in serum, plasma, blood, CSF or tissue; or
5. Detection of a fourfold or greater rise in antibodies specific to JE virus as measured by haemagglutination inhibition (HI) or plaque reduction neutralization assay (PRNT) in serum collected during the acute and convalescent phase of illness. The two specimens should be collected at least 14 days apart and should be done in parallel with other confirmatory tests to eliminate the possibility of cross-reactivity with other flaviviruses such as West Nile and dengue.

Country: Myanmar

JE Campaigns	2006	2007	2008	2009	2010	2011	2012	2013	2014	2015	2016	2017	2018	2019
Target population (under 15 years)*												13.6 million		
Total # of JE doses received in country for campaigns												15,577,470		
Total # children under 15 years who received at least 1 dose of JE vaccine												12.58 million		
Campaign status (national/sub-national/none)												National		
If campaigns were subnational, please list regions where JE campaigns were conducted:														
Type of JE vaccine used (1)**												Live-attenuated (CD-JEV/SA 14-14-2)		
Total # doses of JE vaccine (1) used												12.58 million		
Type of JE vaccine used (2)**														
Total # doses of JE vaccine (2) used														
Type of JE vaccine used (3)**														
Total # doses of JE vaccine (3) used														

***"Target Population (under 15 years)"** refers to the total number of children under 15 years of age who are part of the campaign catchment area(s), regardless if they were vaccinated against JE or not.

****"Type of JE vaccine used (1)-(3)"** There are three data fields for countries that use more than one type of JE vaccine. Use the drop down list to select. If your country only uses one type of JE vaccine, please leave the other data fields blank.

Myanmar

* CD-JEV doses for RI in 2018 are based on cumulative Provincial coverage data provided by WHO

Routine Immunization	2006	2007	2008	2009	2010	2011	2012	2013	2014	2015	2016	2017	2018	2019	2020
Targeted age for routine immunization (RI)													9 months	9 months	9 months
Total target population*													959,076	968376	927884
Total # of JE doses received in country for RI													1,330,100		
Total # children who received at least 1 dose of JE vaccine													853578*	813,436*	807259*
RI status (national/sub-national/none)													National	National	National
If RI is sub-national, please list regions where JE vaccination is part of RI:															
Type of JE vaccine used (1)**													Live-attenuated (CD-JE		C-JEV
# of doses in the primary series for JE vaccine (1)													1	1	1
Total # doses of JE vaccine (1) used													853,578	813,486	807,259
Booster dose for JE vaccine (1)? (Yes/No)															
Type of JE vaccine used (2)**															
# of doses in the primary series for JE vaccine (2)															
Total # doses of JE vaccine (2) used															
Booster dose for JE vaccine (2)? (Yes/No)															
Type of JE vaccine used (3)**															
# of doses in the primary series for JE vaccine (3)															
Total # doses of JE vaccine (3) used															
Booster dose for JE vaccine (3)? (Yes/No)															

***"Total target population"** refers to the total number of children eligible for JE vaccination as part of RI, regardless if they were vaccinated against JE or not.

****"Type of JE vaccine used (1)-(3)"** There are three data fields for countries that use more than one type of JE vaccine. Use the drop down list to select. If your country only uses one type of JE vaccine, please leave the other data fields blank.

Country: Nepal

AES	2006	2007	2008	2009	2010	2011	2012	2013	2014	2015	2016	2017	2018
Population under 15 years	10207313	10434902	10659967	10891703	10936258	11125854	10452459	10000227	10023496	10174478	8676334	8675861	8672396
Total # AES* Cases	1484	1657	1988	1519	1615	1337	952	1227	1404	944	1073	1195	1190
# AES* cases in children (under 15 years)	938	1157	1344	1108	1160	928	630	751	790	544	608	618	611
Total # confirmed[1] JE cases	295	442	339	147	197	128	79	127	226	140	98	88	65
# confirmed JE cases in children (under 15 years)	158	313	234	108	128	69	47	71	112	77	67	47	27

AES = acute encephalitis syndrome which is defined by WHO as a person of any age at any time of year with the acute onset of fever and at least one of the following: 1) a change in mental status (including symptoms such as confusion, disorientation, coma or inability to talk); OR 2) new onset of seizures (excluding simple febrile seizures).

* If your country uses another definition for suspected JE, please give it here:

A *confirmed* JE case according to the WHO's criteria for laboratory-confirmed JE:
1. Presence of IgM antibodies specific to JE virus in a single sample of cerebrospinal fluid (CSF) or serum as detected by an IgM-capture ELISA specifically for JE virus; or
2. Detection of JE-virus antigens in tissue by immunohistochemistry; or
3. Detection of JE-virus genome in serum, plasma, blood, CSF or tissue by reverse transcriptase polymerase chain reaction (PCR) or an equally sensitive and specific nucleic acid amplification test; or
4. Isolation of JE virus in serum, plasma, blood, CSF or tissue; or
5. Detection of a fourfold or greater rise in antibodies specific to JE virus as measured by haemagglutination inhibition (HI) or plaque reduction neutralization assay (PRNT) in serum collected during the acute and convalescent phase of illness. The two specimens should be collected at least 14 days apart and should be done in parallel with other confirmatory tests to eliminate the possibility of cross-reactivity with other flaviviruses such as West Nile and dengue.

Country: Nepal

JE Campaigns	2006 [1]	2007 [1]	2008 [3]	2009 [4]	2010 [4]	2011 [4]	2012	2013	2014	2015	2016 [5]	2017	2018
Target population (under 15 years)*	2,482,678	1,679,806	1,743,666	2,932,381	1,311,590	3,423,133					3,251,435		
Total # of JE doses received in country for campaigns													
Total # children under 15 years who received at least 1 dose of JE vaccine	2,178,062	1,536,980	1,692,764	2,858,084	978,254	2,680,423					3,440,346		
Campaign status (national/sub-national/none)	Sub-National	Sub-National	Sub-National	Sub-National	Sub-National	Sub-National	None	None	None	None	Sub-National	None	None
If campaigns were subnational, please list regions where JE campaigns were conducted:	MWDR, WDR,FWDR	EDR, CDR, WDR, FWDR	EDR,CDR,WDR	EDR, CDR	CDR	CDR, WDR					EDR, CDR,WDR,MDWR, FDWR		
Type of JE vaccine used (1)**	attenuated (CD-JEV/SA 14-14-2)	Live-attenuated (CD-JEV/SA 14-14-2)	attenuated (CD-JEV/SA 14-14-2)	attenuated (CD-JEV/SA 14-14-2)	attenuated (CD-JEV/SA 14-14-2)	attenuated (CD-JEV/SA 14-14-2)					Live-attenuated (CD-JEV/SA 14-14-2)		
Total # doses of JE vaccine (1) used													
Type of JE vaccine used (2)**		-											
Total # doses of JE vaccine (2) used	0%	0%	0%	0%	0%	0%					0%		
Type of JE vaccine used (3)**		-											
Total # doses of JE vaccine (3) used	0%	0%	0%	0%	0%	0%					0%		

***"Target Population (under 15 years)"** refers to the total number of children under 15 years of age who are part of the campaign catchment area(s), regardless if they were vaccinated against JE or not.

****"Type of JE vaccine used (1)-(3)"** There are three data fields for countries that use more than one type of JE vaccine. Use the drop down list to select. If your country only uses one type of JE vaccine, please leave the other data fields blank.

1 - Campign took plance in two phases. Target age group of the camping was above 1 year to under 15 years in the first phase districts and > 15 years in second phase district

Country: Nepal

Routine Immunization	2006	2007	2008	2009	2010	2011	2012	2013	2014	2015	2016	2017	2018
Targeted age for routine immunization (RI)					*12-23 months*	*12-23 months*	*12-23 months*	*12-23 months*	*12-23 months*	*12-23 months*	*12-23 months*	*12 months*	*12 months*
Total target population*					331,013	370,191	419,595	413097	341,165	407303	476693	626022	619197
Total # of JE doses received in country for RI										1008650	not done	not done	not done
Total # children who received at least 1 dose of JE vaccine					154,988	201,277	260,040	296,069	267,738	302291	301466	420494	492017
RI status (national/sub-national/none)	None	None	None	None	Sub-National	Sub-National	Sub-National	Sub-National	Sub-National	Sub-National	Sub-National	National	National
If RI is sub-national, please list regions districts where JE vaccination is part of RI:					22 districts	22 districts	31 districts	31 districts	31 districts	31 districts	31 districts		
Type of JE vaccine used (1)**					Live-attenuated (CD-JE	Live-attenuated (CD-JE	Live-attenuated (CD-JE	Live-attenuated (CD-JE	Live-attenuated (CD-JE	Live-attenuated (CD-JE	Live-attenuated (CD-JE	Live-attenuated (CD-JE	Live-attenuated (CD-JEV/SA 14-14-2)
# of doses in the primary series for JE vaccine (1)					1	1	1	1	1	1	1	1	1
Total # doses of JE vaccine (1) used					154,988	201,277	260,040	296,069	267,738	302,291	301,466	420,494	492,017
Booster dose for JE vaccine (1)? (Yes/No)					No	No	No	No	No	No	No	No	No
Type of JE vaccine used (2)**													
# of doses in the primary series for JE vaccine (2)													
Total # doses of JE vaccine (2) used													
Booster dose for JE vaccine (2)? (Yes/No)													
Type of JE vaccine used (3)**													
# of doses in the primary series for JE vaccine (3)													
Total # doses of JE vaccine (3) used													
Booster dose for JE vaccine (3)? (Yes/No)													

***"Total target population"** refers to the total number of children eligible for JE vaccination as part of RI, regardless if they were vaccinated against JE or not.

****"Type of JE vaccine used (1)-(3)"** There are three data fields for countries that use more than one type of JE vaccine. Use the drop down list to select. If your country only uses one type of JE vaccine, please leave the other data fields blank.

JE & AES Cases

Country: Taiwan

2000 census under 15 years 4,664,891 vs. 2010 census 3,582,513: 23.2% decline in 10 years

AES	2006	2007	2008	2009	2010	2011	2012	2013	2014	2015	2016	2017	2018	2019
Population under 15 years	4145631	4030645	3905203	3778018	3624311	3501790	3411677	3346601	3277300	3187780	3141881	3091873	3048227	3010351
Total # AES* Cases														
# AES* cases in children (under 15 years)	0	0	0	0	0	0	0	0	0	0	0	0	0	0
Total # confirmed[1] JE cases	29	37	17	18	33	22	32	16	18	30	23	25	37	21
# confirmed JE cases in children (under 15 years)	0	0	0	0	0	0	0	0	0	0	0	0	0	0

AES = acute encephalitis syndrome which is defined by WHO as a person of any age at any time of year with the acute onset of fever and at least one of the following: 1) a change in mental status (including symptoms such as confusion, disorientation, coma or inability to talk); OR 2) new onset of seizures (excluding simple febrile seizures).

* If your country uses another definition for suspected JE, please give it here:

A *confirmed* JE case according to the WHO's criteria for laboratory-confirmed JE:
1. Presence of IgM antibodies specific to JE virus in a single sample of cerebrospinal fluid (CSF) or serum as detected by an IgM-capture ELISA specifically for JE virus; or
2. Detection of JE-virus antigens in tissue by immunohistochemistry; or
3. Detection of JE-virus genome in serum, plasma, blood, CSF or tissue by reverse transcriptase polymerase chain reaction (PCR) or an equally sensitive and specific nucleic acid amplification test; or
4. Isolation of JE virus in serum, plasma, blood, CSF or tissue; or
5. Detection of a fourfold or greater rise in antibodies specific to JE virus as measured by haemagglutination inhibition (HI) or plaque reduction neutralization assay (PRNT) in serum collected during the acute and convalescent phase of illness. The two specimens should be collected at least 14 days apart and should be done in parallel with other confirmatory tests to eliminate the possibility of cross-reactivity with other flaviviruses such as West Nile and dengue.

JE Campaigns

Taiwan

No campaigns were conducted with JE vaccine of any type

JE Campaigns	2006	2007	2008	2009	2010	2011	2012	2013	2014	2015	2016	2017	2018
Target population (under 15 years)*													
Total # of JE doses received in country for campaigns													
Total # children under 15 years who received at least 1 dose of JE vaccine													
Campaign status (national/sub-national/none)													
If campaigns were subnational, please list regions where JE campaigns were conducted:													
Type of JE vaccine used (1)**													
Total # doses of JE vaccine (1) used													
Type of JE vaccine used (2)**													
Total # doses of JE vaccine (2) used													
Type of JE vaccine used (3)**													
Total # doses of JE vaccine (3) used													

***"Target Population (under 15 years)"** refers to the total number of children under 15 years of age who are part of the campaign catchment area(s), regardless if they were vaccinated against JE or not.

****"Type of JE vaccine used (1)-(3)"** There are three data fields for countries that use more than one type of JE vaccine. Use the drop down list to select. If your country only uses one type of JE vaccine, please leave the other data fields blank.

** target pop = birth cohort for prior year + 25% - infant mortality using infant mort rate for that year/1000 live births

RI for mbd at 15, 16, 28 months with booster at 5 years

Taiwan

RI for s-p chimeric live vaccine at 15 months and boost is at 27 months-begun in May 2017 as replacement for mbd

JE Routiine immunization

* highest of 15/16 month primary doses + other

Routine Immunization	2006	2007	2008	2009	2010	2011	2012	2013	2014	2015	2016	2017	2018
Targeted age for routine immunization (RI)													
Total target population*	255848**	254168	254137	247124	237942	207582	244653	285588	247845	261893	265923	259528	241380
Total # of JE doses received in country for RI													
Total # children who received at least 1 dose of JE vaccine	*206344	191335	196709	197182	182338	188537	192887	243163	212657	204715	212253	93847	204231
RI status (national/sub-national/none)	National	National	National	National	National	National	National	National	National	National	National	National	National
If RI is sub-national, please list regions where JE vaccination is part of RI:													
Type of JE vaccine used (1)**	Mouse-brain de	Mouse-brain de	Mouse-brain de	Mouse-brain de	Mouse-brain de	Mouse-brain de	Mouse-brain de	Mouse-brain de	Mouse-brain de	Mouse-brain de	Mouse-brain de	Mouse-bra	Live-recombinant (ChimeriVax)
# of doses in the primary series for JE vaccine (1)	3	3	3	3	3	3	3	3	3	3	3	3	1
Total # doses of JE vaccine (1) used	607023	574972	577530	586978	550504	554411	574429	667453	646750	624197	626956	263459	204231
Booster dose for JE vaccine (1)? (Yes/No)	Yes	Yes	Yes	Yes	Yes	Yes	Yes	Yes	Yes	Yes	Yes	Yes	Yes
Type of JE vaccine used (2)**												Live-recombinant (ChimeriVax)	
# of doses in the primary series for JE vaccine (2)												1	
Total # doses of JE vaccine (2) used												134620	
Booster dose for JE vaccine (2)? (Yes/No)												Yes	
Type of JE vaccine used (3)**													
# of doses in the primary series for JE vaccine (3)													
Total # doses of JE vaccine (3) used													
Booster dose for JE vaccine (3)? (Yes/No)													

***"Total target population"** refers to the total number of children eligible for JE vaccination as part of RI, regardless if they were vaccinated against JE or not.

****"Type of JE vaccine used (1)-(3)"** There are three data fields for countries that use more than one type of JE vaccine. Use the drop down list to select. If your country only uses one type of JE vaccine, please leave the other data fields blank.

JE&AES cases

Country: Vietnam

AES	2006	2007	2008	2009	2010	2011	2012	2013	2014	2015	2016	2017	2018	2019
Population under 15 years	20,161,300	20,380,800	20,598,700	20,993,000	20,864,000	20,850,000	20,928,000	21,063,000	21,230,000	21,394,000	21,561,000	21,725,000	21,884,000	21,884,000
Total # AES* Cases (from GDPM data; viral encephalitis)	1550	1208	1345	1227	1115	1273	1138	945	999	940	1043	862	961	
# AES* cases in children (under 15 years)	ND	ND	ND	ND	ND	ND	ND	ND	ND	ND	ND	ND	ND	
Total # confirmed[1] JE cases (from JRF & Annual EPI report = based on 2 National Laboratories data + 3 ME sentinel sites)				68	140	126	183	224	476	368	357	200	313	196**
# confirmed JE cases in children (under 15 years)				9	40	70	163	190	422	347	291	193	278	~170
# confirmed JE cases in children (under 5 years)						17	45	71	154	92	100	65	100	
Total # confirmed JE cases from Thai Binh province as one of the JE sentinel surveillance sites		16		8	15	1	7	7	3	2	4	4	2	
# confirmed JE cases in children from Thai Binh province (under 15 years)				5	8	1	2	3	0	0	2	3	0	

AES = acute encephalitis syndrome which is defined by WHO as a person of any age at any time of year with the acute onset of fever and at least one of the following: 1) a change in mental status (including symptoms such as confusion, disorientation, coma or inability to talk); OR 2) new onset of seizures (excluding simple febrile seizures).

* If your country uses another definition for suspected JE, please give it here: A suspected viral encephalitis case is a case that have sign of acute meningo-encephalitis including: 1) high fever 38-40oC with headache, vomit, convulsion, neck stiffness; 2) Changing in mental status , confusion, disorientation, mobility disorders (convulsion, ...), coma, inability to talk, paralysis.

A *confirmed* JE case according to the WHO's criteria for laboratory-confirmed JE:
1. Presence of IgM antibodies specific to JE virus in a single sample of cerebrospinal fluid (CSF) or serum as detected by an IgM-capture ELISA specifically for JE virus; or
2. Detection of JE-virus antigens in tissue by immunohistochemistry; or
3. Detection of JE-virus genome in serum, plasma, blood, CSF or tissue by reverse transcriptase polymerase chain reaction (PCR) or an equally sensitive and specific nucleic acid amplification test; or
4. Isolation of JE virus in serum, plasma, blood, CSF or tissue; or
5. Detection of a fourfold or greater rise in antibodies specific to JE virus as measured by haemagglutination inhibition (HI) or plaque reduction neutralization assay (PRNT) in serum collected during the acute and convalescent phase of illness.
The two specimens should be collected at least 14 days apart and should be done in parallel with other confirmatory tests to eliminate the possibility of cross-reactivity with other flaviviruses such as West Nile and dengue.

** 196 all age total for 2019 from NIHE. 2011-18 proportion of <15 yrs to all age is 87.0% leading to extrapolated 170 2019 cases for 2019

2009-2018
20,993,000
20,864,000
20,850,000
20,928,000
21,063,000
21,230,000
21,394,000
21,561,000
21,725,000
21,884,000
212,492,000

JE Vaccinaiton Campaigns

Country: Vietnam

JE Campaigns	2006	2007	2008	2009	2010	2011	2012	2013	2014	2015	2016	2017	2018
Target population (under 15 years)*													182,480
Total # of JE doses received in country for campaigns													523,422
Total # children under 15 years who received at least 1 dose of JE vaccine													174474
Campaign status (national/sub-national/none)													Sub-National
If campaigns were subnational, please list regions where JE campaigns were conducted:													28 high risk districts in 16 provinves
Type of JE vaccine used (1)**													Mouse-brain derived
Total # doses of JE vaccine (1) used (ml)													939,870
Type of JE vaccine used (2)**													
Total # doses of JE vaccine (2) used													
Type of JE vaccine used (3)**													
Total # doses of JE vaccine (3) used													

***"Target Population (under 15 years)"** refers to the total number of children under 15 years of age who are part of the campaign catchment area(s), regardless if they were vaccinated against JE or not.
****"Type of JE vaccine used (1)-(3)"** There are three data fields for countries that use more than one type of JE vaccine. Use the drop down list to select. If your country only uses one type of JE vaccine, please leave the other data fields blank.

JE Routine Immunization

Country: Vietnam

Routine Immunization	2006	2007	2008	2009	2010	2011	2012	2013	2014	2015	2016	2017	2018
Targeted age for routine immunization (RI)	1-5 y	1-5 y	1-5 y	1-5 y	1-5 y	1-5 y	1-5 y	1-5 y	1-5 y	1-5 y	1-5 y	1-5 y	1-5 y
Total target population* (dose 1-2)	1,071,493	1,221,362	1,354,526	1,575,730	787,086	1,487,247	1,426,025	1,472,612	1,907,637	2,173,194	1,862,401	1,641,808	1,946,202
Total target population* (dose 3)	837,778	971,457	1,141,554	1,259,266	642,899	1,222,944	1,453,562	1,271,088	1,377,780	1,904,458	1,959,417	1,681,906	1,715,276
Total # of JE doses received in country for RI	2,811,259	3,038,387	3,439,071	4,126,991	2,074,963	3,743,723	4,056,153	3,854,311	4,886,108	5,731,134	5,299,728	4,669,311	5,082,283
Total # children who received at least 1 dose of JE vaccine	1,014,930	1,068,732	1,194,866	1,466,528	728,432	1,346,460	1,338,752	1,336,689	1,786,787	1,980,804	1,764,640	1,548,083	1,780,836
RI status (national/sub-national/none)	Sub-National	Sub-National	Sub-National	Sub-National	Sub-National	Sub-National	Sub-National	Sub-National	Sub-National	National	National	National	National
If RI is sub-national, please list regions where JE vaccination is part of RI:													
Total number of district	673	676	684	689	695	696	697	698	704	707	710	710	710
# districts	372	437	478	532	314	510	566	580	691	707	710	710	710
Type of JE vaccine used (1)**	Mouse-brain de	Mouse-brain de	Mouse-brain de	Mouse-brain de	Mouse-brain de	Mouse-brain de	Mouse-brain de	Mouse-brain de	Mouse-brain de	Mouse-brain de	Mouse-brain de	Mouse-brain de	Mouse-brain derived
# of doses in the primary series for JE vaccine (1)	3	3	3	3	3	3	3	3	3	3	2	3	3
Total # doses of JE vaccine (1) used (ml)	3,115,220	1,848,445	2,295,505	2,701,705	1,296,150	2,840,605	2,418,090	2,447,345	3,514,730	4,151,655	3,540,710	3,189,700	3,757,540
Booster dose for JE vaccine (1)? (Yes/No)	No	No	No	No	No	No	No	No	No	No	No	No	No
Type of JE vaccine used (2)**	None (N/A)	None (N/A)	None (N/A)	None (N/A)	None (N/A)	None (N/A)	None (N/A)	None (N/A)	None (N/A)	None (N/A)	None (N/A)	None (N/A)	None (N/A)
# of doses in the primary series for JE vaccine (2)													
Total # doses of JE vaccine (2) used													
Booster dose for JE vaccine (2)? (Yes/No)													
Type of JE vaccine used (3)**	None (N/A)	None (N/A)	None (N/A)	None (N/A)	None (N/A)	None (N/A)	None (N/A)	None (N/A)	None (N/A)	None (N/A)	None (N/A)	None (N/A)	None (N/A)
# of doses in the primary series for JE vaccine (3)													
Total # doses of JE vaccine (3) used													
Booster dose for JE vaccine (3)? (Yes/No)													

***"Total target population"** refers to the total number of children eligible for JE vaccination as part of RI, regardless if they were vaccinated against JE or not.

****"Type of JE vaccine used (1)-(3)"** There are three data fields for countries that use more than one type of JE vaccine. Use the drop down list to select. If your country only uses one type of JE vaccine, please leave the other data fields blank.

Add clarification of RI introduction strategy in Viet Nam

In 1997, JE vaccination was conducted in 11 epidemiological focused districts of 11 northern provinces with coverage of more than 95% for children <5 years old in these selected districts. In the coming year, the vaccination expanded to other districts/provinces by 2015 (707/707 districts). In the new district, the vaccine will cover children to under 5 years old with 3 dose of basic immunization as scheduled in the national immunization schedule. After at least 4 years of covering to children under 5 years, the vaccine will be only given to children under 36 months old. Each year, JE vaccination will be conducted every 2-3 times/year in each district/province. By 2015, The JE vaccine was intergrated in monthly vaccination day in comune heath center similar to the other EPI vaccine.

JE routine immunization

Country: Thailand

Routine Immunization	2006	2007	2008	2009	2010	2011	2012	2013	2014	2015	2016	2017	2018	2019
Targeted age for routine immunization (RI)	Primary dose in	Primary dose in	Primary dose in	Primary dose in	Primary dose in	Primary dose in	Primary dose in	Primary dose in	Primary dose in	Primary dose in	Primary dose in	Primary dose in	Primary dose in the age of 1 year and booster dose in the age of 2 years and a half	
Total target population*	1,599,689	1,580,029	1,574,256	1,563,933	1,541,296	1,514,196	1,516,611	1,558,522	1,522,482	1,455,387	1,401,812	1,338,697	1,309,396	1,273,483
Total # of JE doses received in country for RI	N/A	N/A	N/A	N/A	N/A	1,600,000	1,524,000	956,218	2,230,000	2,114,000	1,890,000	2,000,000	2,550,000	2,000,000
Total # children who received at least 1 dose of JE vaccine	N/A	N/A	N/A	784,932	786,001	782,902	920,346	819,020	834,677	861,919	828,423	785,617	729,642	744,092
RI status (national/sub-national/none)	National	National	National	National	National	National	National	National	National	National	National	National	National	National
If RI is sub-national, please list regions where JE vaccination is part of RI:														
Type of JE vaccine used (1)**	Mouse-brain de	Mouse-brain de	Mouse-brain de	Mouse-brain de	Mouse-brain de	Mouse-brain de	Mouse-brain de	Mouse-brain de	Mouse-brain de	Mouse-brain de	Mouse-brain derived			
# of doses in the primary series for JE vaccine (1)	2	2	2	2	2	2	2	2	2	2	2			
Total # doses of JE vaccine (1) used	N/A	N/A	N/A	N/A	1,600,000	2,400,000	2,160,000	1,080,000	2,148,000	1,440,000	924,040			
Booster dose for JE vaccine (1)? (Yes/No)	Yes	Yes	Yes	Yes	Yes	Yes	Yes	Yes	Yes	Yes	Yes			
Type of JE vaccine used (2)**								Live-attenuated	Live-attenuated	Live-attenuated	Live-attenuated	Live-attenuated	Live-attenuated	Live-attenuated (CD-JEV/SA 14-14-2)
# of doses in the primary series for JE vaccine (2)								1	1	1	1	1	1	1
Total # doses of JE vaccine (2) used								100,000	126,000	540,000	577,002	821,725	1,287,512	1,056,821
Booster dose for JE vaccine (2)? (Yes/No)								Yes	Yes	Yes	Yes	Yes	Yes	Yes
Type of JE vaccine used (3)**												Live-recombinan	Live-recombinan	Live-recombinant (ChimeriVax)
# of doses in the primary series for JE vaccine (3)												1	1	1
Total # doses of JE vaccine (3) used												718,748	252,924	76,698
Booster dose for JE vaccine (3)? (Yes/No)												Yes	Yes	Yes

***"Total target population"** refers to the total number of children eligible for JE vaccination as part of RI, regardless if they were vaccinated against JE or not.

****"Type of JE vaccine used (1)-(3)"** There are three data fields for countries that use more than one type of JE vaccine. Use the drop down list to select. If your country only uses one type of JE vaccine, please leave the other data fields blank.

Country: Thailand

JE & AES Cases

AES	2006	2007	2008	2009	2010	2011	2012	2013	2014	2015	2016	2017	2018
Population under 15 years	13105098	12924777	12752343	12580529	12360390	12115920	11959619	11804488	11699299	11557397	11433331	11304871	11153397
Total # AES* Cases	273	325	353	372	391	520	591	657	565	622	772	862	879
# AES* cases in children (under 15 years)	146	159	152	141	115	161	154	193	136	128	159	204	184
Total # confirmed[1] JE cases	49	43	64	36	40	52	60	61	11	12	20	14	17
# confirmed JE cases in children (under 15 years)	33	28	34	22	29	27	41	47	8	8	25	23	13

AES = acute encephalitis syndrome which is defined by WHO as a person of any age at any time of year with the acute onset of fever and at least one of the following: 1) a change in mental status (including symptoms such as confusion, disorientation, coma or inability to talk); OR 2) new onset of seizures (excluding simple febrile seizures).

* If your country uses another definition for suspected JE, please give it here:

A *confirmed* JE case according to the WHO's criteria for laboratory-confirmed JE:
1. Presence of IgM antibodies specific to JE virus in a single sample of cerebrospinal fluid (CSF) or serum as detected by an IgM-capture ELISA specifically for JE virus; or
2. Detection of JE-virus antigens in tissue by immunohistochemistry; or
3. Detection of JE-virus genome in serum, plasma, blood, CSF or tissue by reverse transcriptase polymerase chain reaction (PCR) or an equally sensitive and specific nucleic acid amplification test; or
4. Isolation of JE virus in serum, plasma, blood, CSF or tissue; or
5. Detection of a fourfold or greater rise in antibodies specific to JE virus as measured by haemagglutination inhibition (HI) or plaque reduction neutralization assay (PRNT) in serum collected during the acute and convalescent phase of illness. The two specimens should be collected at least 14 days apart and should be done in parallel with other confirmatory tests to eliminate the possibility of cross-reactivity with other flaviviruses such as West Nile and dengue.

Country: Thailand

JE Campaigns	2006	2007	2008	2009	2010	2011	2012	2013	2014	2015	2016	2017	2018
Target population (under 15 years)*													
Total # of JE doses received in country for campaigns													
Total # children under 15 years who received at least 1 dose of JE vaccine													
Campaign status (national/sub-national/none)	None	None	None	None	None	None	None	None	None	None	None	None	None
If campaigns were subnational, please list regions where JE campaigns were conducted:													
Type of JE vaccine used (1)**													
Total # doses of JE vaccine (1) used													
Type of JE vaccine used (2)**													
Total # doses of JE vaccine (2) used													
Type of JE vaccine used (3)**													
Total # doses of JE vaccine (3) used													

***"Target Population (under 15 years)"** refers to the total number of children under 15 years of age who are part of the campaign catchment area(s), regardless if they were vaccinated against JE or not.

****"Type of JE vaccine used (1)-(3)"** There are three data fields for countries that use more than one type of JE vaccine. Use the drop down list to select. If your country only uses one type of JE vaccine, please leave the other data fields blank.
