# Supplementary material for: Impact of vaccination against Japanese encephalitis in endemic countries
Source: PLoS Negl Trop Dis. 2024 Sep 3;18(9):e0012390. doi: 10.1371/journal.pntd.0012390 (PMC11398676; doi:10.1371/journal.pntd.0012390)
Supplement: S1 Acknowledgements — (DOCX) [file pntd.0012390.s004.docx]

**The** **JE Vaccine Global Impact Assessment Team**

**Members of the JE Vaccine Global Impact Assessment Team:** Bangladesh-Rajendra Bohara WCO ([boharar@who.int](mailto:boharar@who.int)), Nihal Abeysinghe (PATH, nihal.ird@gmail.com); Bhutan-Tshewang Dorji Tamang EPI (ttamang@health.gov.bt), Sonam Yangchen WCO (yanchens@who.int); Cambodia-Siphan Sovarnara NIP ([siphan_sovannara@yahoo.com](mailto:siphan_sovannara@yahoo.com)); China, People’s Republic-Wu Dan China CDC ([Wudan@chinacdc.cn](mailto:Wudan@chinacdc.cn)) Yuan Yuan (PATH Beijing China yyuan@path.org); Chinese Taipei-Chwan-Chuen King MOH ([chwanchuen@gmail.com](mailto:chwanchuen@gmail.com)); India-Pradeep Haldar EPI ([pradeephaldar@yahoo.co.in](mailto:pradeephaldar@yahoo.co.in)), Shalini Khare (PATH Dehli India, drshalz@rediffmail.com); Indonesia- Hashta Meyta MOH, Vinod Bura WCO ([burav@who.int](mailto:burav@who.int)), Justin Im International Vaccine Institute (IVI) Seoul Korea ([justin.im@rightfoundation.kr](mailto:justin.im@rightfoundation.kr)) Andrea Haselbeck [(andreahaselbeck@gmail.com](mailto:(andreahaselbeck@gmail.com)) IVI-Bali; Japan-Mayasuki Saijo National Institute of Infectious Diseases ([msaijo@nih.go.jp](mailto:msaijo@nih.go.jp)); Democratic People’s Republic of Korea (North Korea)-Md. Rezwan Kamar WCO (rezwank@who.int); Republic of Korea-Taeun Yang Korean CDC ([taeun.yang@gmail.com](mailto:taeun.yang@gmail.com)); Laos-Chansay Pathammavong EPI ([chansay_epi@yahoo.com](mailto:chansay_epi@yahoo.com)); Malaysia-Jeffery Stephen CDC Sarawak ([stjeffery1974@gmail.com](mailto:stjeffery1974@gmail.com); Myanmar-Stephen Chacko WCO (chackos@who.int); Nepal- Abhiyan Gautam MOH (gautamabhiyan@gmail.com), Rajendra Prasad Pant MOH ([rajendrapant8@gmail.com](mailto:rajendrapant8@gmail.com)), Anindya Bose WCO (bosea@who.int); Papua New Guinea-Deborah Bettels WCO (bettelsd@who.int); Philippines- Achyut Shreshtha WCO (shresthaa@who.int); Singapore-Chun Paul Soo WCO ([sooc@who.int](mailto:sooc@who.int)); Sri Lanka-Purna Samitha Ginige MOH (samithag@hotmail.com); Thailand-Chaninan Sonthichai MOH ([chaninan33@yahoo.com](mailto:chaninan33@yahoo.com)), Pimpa Techakomolsuk MOH ([t.pimpa@gmail.com](mailto:t.pimpa@gmail.com)), Aree Moungsookajareaoun WCO (aree@who.int); Timor leste-T Sudath R Peiris WCO (peirist@who.int); Makiko Iijima WCO Vietnam (iijimam@who.int); Jayantha Lyanage WHO Immunizations Director, Southeast Asia Region, Dehli India (liyanagej@who.int); James Heffelfinger WHO Immunizations Director, Western Pacific Region, Manila Philippines (WHO liaison for Australia, Brunei, Peoples Republic of China, Japan, Laos PDR and Republic of Korea) (heffelfingerj@who.int).

**All members of JE Global Impact Assessment Team contributed refined data for analysis according to protocol and were involved in the refinement, stratification and analysis of the data acquired by submitted questionnaire or presented to the BiRegional meetings. Jayantha Liyanage and James Heffelfinger contributed to the design of the study in addition to facilitating data acquisition.**

Abbreviations:(CDC) country Centers for Disease Control and Prevention; (EPI) Expanded Program Manager for Immunizations in country MOH; (MOH) country Ministry of Health; (NGO) non-government organization; (NIP) National Immunization Program in country MOH; (JE) Japanese encephalitis; (WCO) WHO country office; (WHO) World Health Organisation.
