# Supplementary material for: Impact of vaccination against Japanese encephalitis in endemic countries
Source: PLoS Negl Trop Dis. 2024 Sep 3;18(9):e0012390. doi: 10.1371/journal.pntd.0012390 (PMC11398676; doi:10.1371/journal.pntd.0012390)
Supplement: Attachment — Submitted filename: PLoS NTD reviewer response letter.docx [file pntd.0012390.s005.docx]

Michael W Gaunt, PhD	February 16, 2024
Academic Editor
PLOS Neglected Tropical Diseases

Andrea Marzi
Section Editor
PLOS Neglected Tropical Diseases

Dear Dr. Gaunt and Ms. Marzi,

Please find enclosed our response to reviewer’s comments on a recently submitted article to PLoS NTD entitled “**Impact of vaccination against Japanese encephalitis in endemic countries”.**

I have uploaded Figure 1 into your PACE platform as requested. As noted in my original submission, the raw data excel files and BiRegional power points that constitute data gathered from countries are held in a repository (JEVISP) in a Box file folder at PATH that can be accessed on request to PATH. The PATH-WHO protocol and IRB permissions can also be found and accessed in this file folder.

**Response to Reviewer 1 comment in single space italics:** *SIAs was not spelled out in the discussion session in the first instance*.

- We have placed spelled out definition of acronym SIA in its first use in Discussion in paragraph six, line 342 as requested. Reviewer 1 had no other requests.

**Response to Reviewer 2 comments in single space italitc;** *Table 1 – suggest putting the info in the superscript “2” in the table – ie “incidence/100,000 under 15 population*”

- We have added incidence/100,000 population to the Incidence boxes on the Tables.

*The authors have pointed out the major weakness being the variable source of the data. They have not elaborated much on what effect that might have had on their conclusions, if any. I suggest adding a comment on this to the discussion*.

- We have outlined our response to request for assessment of the multiple sources data effect on analysis in a yellow highlighted sentence in lines 367-9 of the limitations first paragraph.

*The authors point out that an estimated 100,000 deaths could be averted by JE vaccination between 2021 and 2030. Could they add a comment to the manuscript on whether their own findings support or refute this?*

- Response to the query as to how we feel our data comment on a possible ~100,000 lives saved between 2021-2030, We have yellow highlighted a reasonable speculation as to the possibility of achieving the VIMC estimate of 100,000 deaths due to JE reduced between 2021-2030 at the end of the next to last paragraph of the Discussion in lines 398-402.

**Response to Reviewer 3 (Methods- in order of reviewer’s comments in single space talics):**

*I believe that the first three bullets in the methods section are meant to represent an overview of the methods that follow but this is not clear as written, particularly since the bullets don’t include the same text that is used for the section headings that follow. Consider adding/editing language to clarify for the reader. Also, these sections include some duplication of information (in main text and bullets) and would be useful to streamline*.

- We have added clarifying terms to bullets and some clarifying language in sections related to bullets below, all in yellow highlight (lines 129, 131, 134 and 168-77).

*The methods for determining vaccine coverage don’t correspond to the data collected through the survey. For example, the survey collected data on the number of doses of vaccine given, but the coverage suggests a list of all children along with their vaccination status. Where did those data come from? Other population-based surveys?*

- We have highlighted clarifying language under description of WHO-PATH survey section, lines 137-9 and 142.

*Methods for Thailand and Vietnam need not be in parentheses as these are important details for the reader*. Additional details for these methods would be useful to include: “We present data for u15 JE incidence in the after-vaccine introduction period to validate use of AE/viral encephalitis reduction in these countries as a estimator of JE as a principal cause of AE/viral encephalitis in these countries.”

- We have set methods for Thailand and Vietnam separately and without parentheses in lines 152-6, plus added some clarifying language in yellow highlight to explain the value of using after vaccine introduction laboratory confirmed surveillance data in the under 15-year population of these countries.

*The statement following statement would benefit from additional clarifications – were these the definitions of a confirmed case for countries? Perhaps this point is better addressed in the discussion rather than the methods? “Because most of the reported JE incidence data are based on the serologic results of serum IgM ELISA, [23] the impact results reported here are considered to be the reduction of probable JE cases.”*

- We have left comment on serum IgM ELISA [23] in methods as it seems to us that the statement is important in pointing out that some would consider our case analysis as based on “probable cases,” however, a sentence describing how countries use serum IgM as “confirmatory” and why is added to this section in lines 179-80. Here also, it still seems to us that this belongs in Methods.

*I presume that the calculations were made for all age groups or for those under the age of 15 depending on the data available from the countries, correct?*

- Yes, this is correct, see lines 168-70

*The following statement is not clear – were these data for Sarawak or Malaysia nationwide? “…and from Sarawak (Malaysia), data represent impact from introduction primarily in Sarawak state and in Maylasia nationwide.”*

- We have added explanation of the analytic situation in Malaysia where immunization was population based only in Sarawak but data was available for comparisons for both Sarawak province and the entire country. This is highlighted in the text lines 189-91.

*The following methods are not quite clear. Can authors add more details? Why was 90% chosen? Also, Figure 1 does not appear to present a rolling average of all the countries studied.*

- We have clarified that 90% reduction was a target chosen based on what Figure 1 shows is possible in countries with long-standing and sustained efforts at JE vaccination. This is highlighted in the Methods describing this side analysis for those early introducing countries (lines 203-5. The reviewer is correct, these line graphs do not represent rolling averages and that language has been removed.
- **Response to Reviewer 3 (Results- in order of reviewer’s comments in single space italics):**

*The pre-introduction study years frequently include the year that the vaccine was introduced, and in some instances, includes many years after the introduction. It’s unclear why these years are termed ‘pre-introduction’. Certainly, this could explain why the incidence change is low? For ROK and Malaysia the time periods are distinct.*

- We have added a clarifying statement in Methods (lines 172-7) where before and after vaccination impact assessment is described as it seems that this is where this belongs even though the reviewer raises it in comment on Results. The reader is also referred to the Appendices which have much greater detail in explanation of specifically why we felt use of some years for assessment of after vaccine introduction were chosen. This was a necessity due to some countries setting up surveillance very close to vaccine introduction. This results in the “pre vaccination” period in some countries really being a period of partial vaccine introduction compared to a much larger or national introduction later. We therefore agree with the reviewers’ comment that this may have reduced the overall impact, most especially in India. We have added country specific comment on that to the discussion in the paragraph on limitations in lines 386-93.

*In the titles of tables, please include details about the age groups and countries that are represented to ensure the table can stand alone.*

- We feel that the Titles reflect exactly that but hope that use of the reviewer’s suggested dropping u15 in favor of “under 15 years” may make the Title easier to read. Although some countries appear in each Table, it is because those countries had available data for both all ages and for under 15 years. It is the age demographic analyzed that distinguishes the Tables.

*For the measured decreases in incidence many years after the introduction of vaccine (Thailand and Vietnam) the ability to infer declines as solely the result of vaccination is greatly reduced. Other social and environmental changes could also be relevant, particularly given the rapid social and ecological changes in these countries over these time periods.*

- We have addressed the comment on changes in social and ecological changes over time in Thailand and Vietnam in Discussion under limitations (lines 379-84) but also have added data in Results to demonstrate that trends were consistently downward over time since vaccine introduction (lines 246-8 for Thailand and 252-4 for Vietnam. Additionally, we point out earlier in Discussion that although sociological or ecological changes should also have reduced dengue where no vaccine effect is yet calculable, dengue incidence trends tend to be upwards although mortality and age distribution have changed over decades (lines 278-91).

*For the countries that did achieve 90% reductions, were other differences also part of why it took many years for JE incidence to decline? I also presume that the type of vaccination campaign would have a large impact on timing – for example, if the campaign only vaccinated infants, it would take 15 years for the highest risk group to all be covered. This additional context seems important to highlight.*

- The reviewer is correct in this query. As we note in the text and Appendices S1 and S2, the early adopting countries used mostly RI in infants and toddlers for introduction and the long time to reduction shown in Figure 1 reflects that.

*Overall, making the link between impact, especially over longer time periods, would be bolstered by showing the increases in vaccination coverage over time to explain trends.*

- We address the comment of the reviewer r.e. vaccine coverage. In Thailand and Vietnam, the coverage has been consistently high since early after vaccine introduction as noted in the section under limitations. The reviewer’s point is important as we note consistent high coverage in most countries with early and sustained JE vaccine introductions as a distinguishing factor between those programs and the younger programs. We refer to the Appendices where these relationships can be seen for most countries. We have added text to point out that long standing programs have sustained high RI coverage whereas in the more recent JE vaccine adopters tend to have RI coverage between 50-70%, even after a decade or more of effort. This information is added to the Discussion in lines 313-17. The peak coverages are shown in the Tables to make just this point for the reader. We clarify in this section of Discussion that those countries achieving >85-90% coverage have done so for many years (see Appendices) but the lower coverage countries have achieved such high coverage only with SIAs and not RI for the most part.

**Response to Reviewer 3 (Conclusions- in order of reviewer’s comments in single space italics):**

The emphasis on pre versus post 2006 seems a bit strange. What is the rationale for this? It’s clear that time since introduction is important to note (as is the target of the vaccine campaigns – see above – but additional rationale for why 2006 was a major turning point would help the reader.
As noted above, could ecological context explain some changes that occurred over 30 years? For example, increased urbanization and contact with vectors?

- To address the first part of the reviewer’s comment: In the second paragraph of the Introduction in the original submission, we noted that in 2006 WHO pronounced vaccination against JE as a public health priority. That led to development of AES and JE surveillance guidelines along with guidelines for introduction of vaccination, a comment we have added to that section for clarity in lines 100-1 and in Discussion, lines 273-4. The second comment on social and ecological changes is addressed in the discussion under limitations in the context of the reviewer’s comments specifically on Thailand and Vietnam, but we have additionally added a comment and reference that addresses the parallel with dengue across the Asia endemic region to the second paragraph of the Discussion lines 278-91.

*The text refers to some of the countries being low-income countries, but I think they’re all at least lower middle income, no? I suppose there is a difference between the country’s economic status when the introduced vaccination and the status today but all references about economic categories report one single category. Perhaps worth discussing any changes over time that might be relevant to the story.*

- Regarding the designation of low vs. low-middle-income, the reviewer is correct that this has been changing over the time of the study. We use the broad LLMIC to encompass all of the current classifications and focus more on the issues of how JE vaccination effort needs to be strengthened regardless of the current economic designation. This is particularly relevant in our discussion of the difficulties in rural areas where logistics are equally difficult whether a country is low or low middle income.

*What is the evidence supporting the claim that the virus might become more highly endemic in some Asian countries? Would be good to cite support.*

- Possible appearance or increase in relative low transmission areas of JE endemicity is supported by established formal AES surveillance in Bangaldesh (data not shown in this paper as Bangladesh had not yet made decision to vaccinate against JE) and by evidence added to the first paragraph of the lines 278-91 of Discussion regarding the spread and/or new regional occurrence of dengue, chikungunya and zika viruses.

**Minor issues**: These are addressed above for Reviewers 1 and 2. The comments here are in response to Reviewer 3 in single space italics:

*Check for typos – no line numbers made it difficult to mention specific instances.

“u15” is not a standard acronym so suggest avoiding.

Best to avoid sentence construction using “respectively” as it requires the reader to go back and re-read the sentence to understand what is being said. 

The abstract would benefit from some edits to clarify a few key issues:

“…in children younger than 15-years-old and in persons of any age…” Can shorten to just say persons of any age since this will cover the range.
Can authors clarify how many endemic countries there are in the abstract? Only 13 or is this a subset? (Methods section methods 23?)
What were the three data collection methods used in the study?
How long was impact measured following vaccination? In Thailand and Vietnam, what were the reductions? Please provide the data to support the main conclusions.
What would there be a difference in impact for countries that introduced before and after 2006? This is unclear.*

- Line numbers have been added and typos corrected where found
- U15 has been changed to under 15 years throughout
- Respectfully disagree with this one and have not changed
- Changes in abstract have been made in yellow highlight, lines 49,50, 53-58,63 and 67

Respectfully submitted

G William Letson MD, MS, FAAP
