# Supplementary material for: Impact of vaccination against Japanese encephalitis in endemic countries
Source: PLoS Negl Trop Dis. 2024 Sep 3;18(9):e0012390. doi: 10.1371/journal.pntd.0012390 (PMC11398676; doi:10.1371/journal.pntd.0012390)
Supplement: Attachment — Submitted filename: PLoS NTD minor revisions reviewer response letter_final.docx [file pntd.0012390.s006.docx]

Michael W Gaunt, PhD	July 16, 2024
Academic Editor
PLOS Neglected Tropical Diseases

Andrea Marzi
Section Editor
PLOS Neglected Tropical Diseases

Dear Dr. Gaunt and Ms. Marzi,

Please find enclosed our response to reviewer comments for our submitted article entitled “**Impact of vaccination against Japanese encephalitis in endemic countries”.** We have removed highlight from the last submission and highlight now reflects the changes detailed below.

In addition, we would like to note that with our initial attempt at submitting response to this request for minor revision on June 20, PLoSNTD editorial staff raised concern about the Biregional power point data files that we had made available as part of our raw data for portions of the study, might be problematic with regard to possible personal identifying pictures and confidential matter. We agreed with those editors and in fact had not submitted this information for that reason. In discussion with your Peer Review Specialist Emy Janelli, JD on June 24-25, 2024 we reached agreement to withdraw this part of our raw data and to offer readers opportunity to request non-identifying and non-confidential data directly from PATH. Raw data gathered according to the WHO-PATH protocol is now listed in the legend after References as S3 and will be immediately available to readers as it contains no identifiers. The delay between June 25 and now was due to securing the proper contact avenue for request of the removed raw data file from PATH. That is now provided in the Data Availability section of the submission and under description of S3 in the legend.

We first address comments of reviewers 1 and 2 as they are few:

Reviewer 1: We have moved description of the acronym SIA to first use as requested line 235). (We have also corrected spelling on variability and removed the out of place parenthetical on lines 275 and 289 respectively.

Reviewer 2: typo on line 287 is corrected and in response to both reviewers 2 and 3 we have gone through the text and made consistent use of the term under age 15 years wherever there was reference to this population. All of these changes are yellow highlighted.

Reviewer 3: responses are ordered according to journal section heading

**Methods:**

Comment on page 10 regarding percentage of AE due to JE: We have added a comment on the percentage of JE among all acute encephalitis cases to go with the earlier cited references (line 145) and moved the paragraph to the end of Methods as suggested by the reviewer. (Note: To match the order of presentation of information in the Methods, we have also changed the order of the “Results” section. The paragraph on impact assessment using acute encephalitis and viral encephalitis data has been moved to the end of the Results section).

**Results:**

Table 1: Reviewer requests additional information on pre-introduction years, whether campaigns were one-time activities, how coverage might be >100% etc: In the appendices, for each country, we have provided data that address all these issues and additional details. To ensure this is clear and the information is accessible to readers, we have added a comment in the Methods section noting “Complete information on pre- and post-introduction study periods and immunization program details for each country is provided in Appendices S1 and S2” (lines 127-128), and added footnotes to Tables 1 and 2 noting “Additional information for each location on rationale for selection of pre- and post-introduction study periods, vaccine coverage estimates, and data sources are provided in Appendices S1 and S2.”

**Conclusions:**

Reviewer requests removal of discussion of other mosquito transmitted arboviruses. We have done this and confined our discussion of this point to JE alone using references on JE disease expansion into new areas within noted endemic countries and to countries without prior JE disease (lines 284-9). This is addressed further in our response to the reviewer’s request that we supply evidence for JE possibly becoming more endemic (lines 213-217). We have noted at lines 296-298 that in Pakistan and parts of India and Bangladesh, confirmed cases have not recently been reported but there is high environmental suitability for *Culex tritaeniorhynchus,* suggesting the potential for increased virus transmission [41-42].

Reviewer asks if we can provide some analysis of how estimates based on the VIMC models compare to our results. If we assume the 69,000 annual estimated cases of JE prior to the 2006-2018 JE vaccine introduction initiative, a median of the estimated 20-30% fatality (25%) and use the median JE reduction shown in Table 1 for countries introducing vaccine (55%), one can estimate the potential impact of this level of reduction on mortality over the 10 years from 2021-30: (69,000)(10)(.25) = 172,500 deaths. Fifty-five percent reduction in this figure would be 94,875 deaths averted, a figure that offers some support to the validity of the VIMC modelled estimate. However this calculation is dependent on major assumptions such as continued JE immunization sustenance, no further improvement, and equal contribution to mortality reduction in each of these countries. Many of these assumptions are likely untrue. More precise comparisons would require extensive analysis and modelling efforts which are beyond the scope of this paper and would also necessarily need to include multiple assumptions about future vaccination efforts and coverage. Given these substantial limitations, we think the included comment is sufficient as noted at lines 301-303: “If JE vaccination is administered in recent and new adopting countries with high integrity of administration as was accomplished in China, Chinese Taipei, Korea and Japan, it is conceivable that the VIMC estimate of a reduction of 100,000 deaths due to JE could be achieved between 2021-2030 [11], assuming the over 90% reduction of JE reported in the early JE vaccine introducers.”

**Editorial and Data Presentation Modifications:**

Reviewer requests a date clarification in Author Summary. We have accordingly changed that to 2006 as requested (line 56).

**Abstract:**

Reviewer requests addition of final year or period after introduction that results relate to and states that the final sentence in Methods and Findings is confusing and asks for some indication in the abstract about why these results are reported differently: We have revised the abstract to incorporate this information and made clarifications, while ensuring we adhere to the maximum 300-word limit for the abstract.

Respectfully submitted

G William Letson MD, MS, FAAP
